# Efficacy and safety of current medications for treating severe and non-severe COVID-19 patients: an updated network meta-analysis of randomized placebo-controlled trials

**DOI:** 10.18632/aging.203522

**Published:** 2021-09-16

**Authors:** Qinglin Cheng, Junfang Chen, Qingjun Jia, Zijian Fang, Gang Zhao

**Affiliations:** 1 Hangzhou Center for Disease Control and Prevention, Hangzhou 310021, China; 2 School of Medicine, Hangzhou Normal University, Hangzhou 310021, China

**Keywords:** efficacy, safety, COVID-19, network meta-analysis, randomized placebo-controlled trials

## Abstract

Background: Many recent studies have investigated the role of drug interventions for coronavirus disease 2019 (COVID-19) infection. However, an important question has been raised about how to select the effective and secure medications for COVID-19 patients. The aim of this analysis was to assess the efficacy and safety of the various medications available for severe and non-severe COVID-19 patients based on randomized placebo-controlled trials (RPCTs).

Methods: We did an updated network meta-analysis. We searched the databases from inception until July 31, 2021, with no language restrictions. We included RPCTs comparing 49 medications and placebo in the treatment of severe and non-severe patients (aged 18 years or older) with COVID-19 infection. We extracted data on the trial and patient characteristics, and the following primary outcomes: all-cause mortality, the ratios of virological cure, and treatment-emergent adverse events. Odds ratio (OR) and their 95% confidence interval (CI) were used as effect estimates.

Results: From 3,869 publications, we included 61 articles related to 73 RPCTs (57 in non-severe COVID-19 patients and 16 in severe COVID-19 patients), comprising 20,680 patients. The mean sample size was 160 (interquartile range 96–393) in this study. The median duration of follow-up drugs intervention was 28 days (interquartile range 21–30). For increase in virological cure, we only found that proxalutamide (OR 9.16, 95% CI 3.15–18.30), ivermectin (OR 6.33, 95% CI 1.22–32.86), and low dosage bamlanivimab (OR 5.29, 95% CI 1.12–24.99) seemed to be associated with non-severe COVID-19 patients when compared with placebo, in which proxalutamide seemed to be better than low dosage bamlanivimab (OR 5.69, 95% CI 2.43–17.65). For decrease in all-cause mortality, we found that proxalutamide (OR 0.13, 95% CI 0.09–0.19), imatinib (OR 0.49, 95% CI 0.25–0.96), and baricitinib (OR 0.58, 95% CI 0.42–0.82) seemed to be associated with non-severe COVID-19 patients; however, we only found that immunoglobulin gamma (OR 0.27, 95% CI 0.08–0.89) was related to severe COVID-19 patients when compared with placebo. For change in treatment-emergent adverse events, we only found that sotrovimab (OR 0.21, 95% CI 0.13–0.34) was associated with non-severe COVID-19 patients; however, we did not find any medications that presented a statistical difference when compared with placebo among severe COVID-19 patients.

Conclusion: We conclude that marked variations exist in the efficacy and safety of medications between severe and non-severe patients with COVID-19. It seems that monoclonal antibodies (e.g., low dosage bamlanivimab, baricitinib, imatinib, and sotrovimab) are a better choice for treating severe or non-severe COVID-19 patients. Clinical decisions to use preferentially medications should carefully consider the risk-benefit profile based on efficacy and safety of all active interventions in patients with COVID-19 at different levels of infection.

## INTRODUCTION

Coronavirus disease 2019 (COVID-19) is an unprecedented global life-threatening pandemic. COVID-19 has generated an enormous public health crisis in the world [1]. Though COVID-19 has a relatively low mortality rate, it can cause a highly lethal rate in high-risk patients [2, 3]. So far, it is unclear how a specific, effective, and secure therapy for severe or non-severe COVID-19 infection is selected [4]. Hence, it is mandatory to identify potential, accurate treatments for patients with severe or non-severe COVID-19 infection [4].

In the past year, pharmacological interventions [e.g., ivermectin, avifavir, doxycycline, sarilumab, bamlanivimab, colchicine, monoclonal antibody, lopinavir/ritonavir (LPV/r), convalescent plasma (CP)] have been widely used in the treatment of COVID-19 patients [4]. A large amount of time and resources have been put into the development of direct-acting antivirals for the SARS-Coronavirus-2 since December 2019 [5]. However, to date, large-scale randomized controlled trials are not only missing due to considering ethics involved but likely also because little time has passed since the emergence of SARS-Coronavirus-2. We did not yet know what drug was the best choice for severe or non-severe COVID-19 patients in clinical practice [5, 6].

How to solve the security and efficacy issues in the therapy of COVID-19 infection has become one of the most important challenges [4–6]. Fortunately, network meta-analysis (NMA) can be helpful in assessing the comparative efficacy and safety of multiple interventions, even if they have not been researched head-to-head in randomized controlled trials [7]. Although previous studies of NMA have been carried out on the treatment interventions of COVID-19, most of these studies might have potentially biased results due to lack of standardizing pharmaceutical interventions or the controls [8, 9]. For instance, we compared the efficacy and safety for standard of care (SOC), which existed the bias due to the differential SOC of every country (i.e., SOC was not standardized) [8, 9]. Additionally, we might not verify the pure efficacy and safety of pharmacological interventions due to a positive control drug [10]. There is paucity of head-to-head randomized placebo-controlled trials (RPCTs) comparing different pharmacological interventions for severe or non-severe COVID-19 patients, which can inform clinicians regarding the comparative efficacy and safety of these interventions based on the degree of COVID-19 infection.

To fill this gap, we did an updated network meta-analysis of RPCTs in current medications with severe or non-severe COVID-19 infection, using all available data from published clinical trials. We aimed specifically to compare the efficacy and safety of medications available for severe or non-severe COVID-19 patients based on RPCTs.

## MATERIALS AND METHODS

Our study was arranged in line with PRISMA (Preferred Reporting Items for Systematic Reviews and Meta-Analyses) guidelines and its extension statement for NMAs [11].

### Data source and search strategy

We searched the relative data for RPCTs of medications recommended for patients with COVID-19 infection in PubMed, Elsevier Science Direct, Cochrane Library, Google Scholar, SpringerLink, MedRxiv, China National Knowledge Infrastructure, and Wanfangdata. The publication date was set from the beginning of 2019 to July 31, 2021, and no language restrictions. Full search strategies were listed in the Supplementary Materials (Appendix 1). We extracted data on RPCTs, patient and therapy drugs characteristics (Supplementary Table 1).

**Figure 1:**
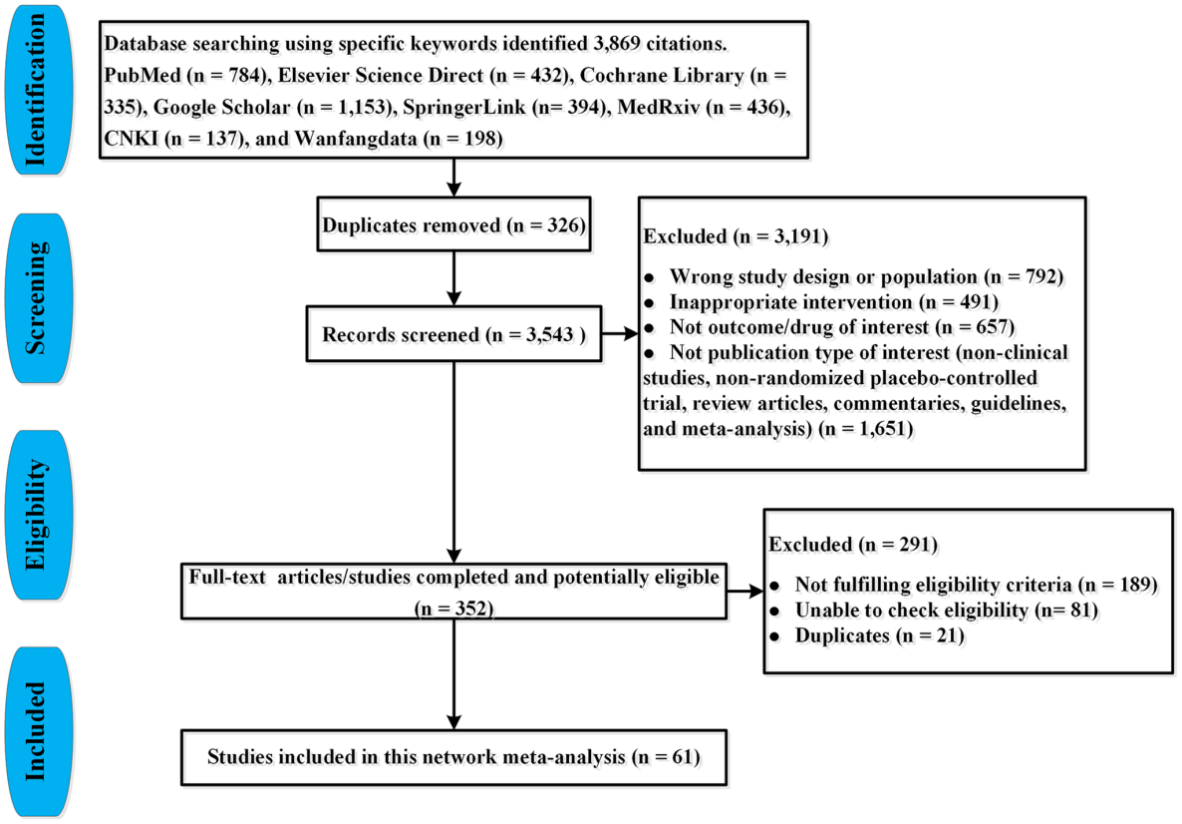
PRISMA flow-chart for study selection.

Two investigators (JQJ and FZJ) via the search strategy screened literature and extracted data. We manually reviewed the titles and abstracts to select the potentially relevant articles’ abstracts and full-texts systematically and comprehensively. Then we carefully read the full-texts and selected eligible articles. Finally, we included all comparative RPCTs for the treatment of COVID-19 patients. The PRISMA flow chart is shown in Figure 1.

### Selection criteria

RPCTs, of at least 1 week’s duration, including adult patients (aged ≥ 18 years) with COVID-19 infection who were in accord with the diagnostic guidelines of World Health Organization [12] were eligible for inclusion. All RPCTs studies that estimated the efficacy or safety between pharmacological interventions and COVID-19 infection were considered for inclusion. Studies were ultimately included if they: (a) were a RPCT; (b) were COVID-19 patients aged 18 years and older; (c) reported COVID-19 related therapy methods as a predictor of clinical outcomes (efficacy or safety), including the ratio of virological cure (VC) or/and all-cause mortality (ACM), or/and treatment-emergent adverse events (TEAEs); and (d) reported any one of the following statistics: VC, ACM, and TEAEs, or other statistics that could be converted into a standardized effect size. Studies were excluded if they: (a) were wrong study design or population (i.e., patients with mild to severe or moderate to severe COVID-19 infections); (b) were duplicated research or not full-text articles; (c) had no outcomes/drugs of interest; (d) reported the publication types of non-clinical studies, non-randomized controlled trial, review articles, commentaries, guidelines, and meta-analysis; (e) no primary or missing data existed after contacting authors; or (f) had considerable heterogeneity of studies’ groups. We resolved any ambiguity through mutual discussion and consensus during selecting eligible studies.

### Data extraction and quality assessment

Two of five investigators (CQL, CJF, FZJ, JQJ and ZG) independently selected eligible studies, reviewed the main data and supplementary materials, extracted the relevant data information from the included RPCTs, and assessed the risk of bias (κ range for interrater reliability 0.81–0.93) by using a standardized form. We extracted the following data from articles that met the criteria: (1) author name (reference); (2) publication year; (3) country/countries of origin; (4) study design; (5) method of COVID-19 testing; (6) patient population; (7) numbers of participants; (8) gender; (9) age; (10) interventions; (11) treatment medication dose; (12) controls; (13) control medication dose; (14) follow-up time (days); and (15) primary outcomes. One investigator undertook the initial extraction of studies, and another reviewed the extraction. Any discrepancies were resolved by discussion and consultation by a panel of researchers within the review team (CQL, CJF, FZJ, JQJ and ZG).

Three investigators (CQL, JQJ and FZJ) assessed the risk of bias for all study designs. We used the Cochrane Risk-of-Bias Tool [13] to evaluate the studies’ risk of bias. We estimated the confidence of evidence contributing to each network estimate using the Grading of Recommendations Assessment, Development, and Evaluation [14].

### Outcome measures and definitions

Our primary outcomes were efficacy (ACM and VC) and safety (TEAEs) between the beginning of intervention and end of follow-up. When the ACM for severe or non-severe COVID-19 patients was measured with the proportion of death due to any cause from treatment initiation to end of follow-up. The VC ratio for severe or non-severe COVID-19 infection was defined as the rate of negative reverse transcription-polymerase chain reaction result at the end of the study. Additionally, the TEAEs ratio for severe or non-severe COVID-19 patients referred to the proportion of any TEAEs from the beginning to the end of the study. Patients with COVID-19 infection were stratified into two groups [15]: (1) non-severe COVID-19 patients including mild and moderate cases (i.e., mild cases represented patients with uncomplicated upper respiratory tract viral infection, and moderate cases represented patients with pneumonia but without need for supplemental oxygen); and (2) severe illness represented patients with fever or suspected respiratory infection, plus one of the following: respiratory rate > 30 breaths/min, severe respiratory distress, or SpO2 ≤ 93% on room air.

### Data synthesis and analysis

#### Assessment of the transitivity assumption

Transitivity is the key underlying assumption of NMA and indirect comparisons. To estimate the transitivity assumption, we investigated the distribution of potential effect modifiers. Possible effect modifiers included multicenter study (MS), duration of study (DS), double blind (DB), crossover design (CD), sample size (SS), industry sponsorship (IS), inequalities in doses (ID), and risk of reported bias (RRB).

### Network meta-analysis

We used STATA statistical software (Version 15, Stata Corporation, and College Station, Texas, USA) and R software version 4.0.4 to perform our Bayesian NMA. Additional details were described in the Supplementary Materials (Appendix 2). Statistical significance was defined as a 2-sided *P*-value of less than 0.05.

To describe the comparative efficacy and safety of all medications, we conducted a Bayesian NMA using all available pharmacological regimens. The NMA provided better comparative evidence than conventional meta-analysis due to the merged applying of direct (e.g., head-to-head comparative studies) and indirect evidence (i.e., single arm and non-comparative studies) or different indirect evidence [16]. We calculated summary odds ratio (OR) with 95% confidence interval (CI) to estimate dichotomous outcomes. The rank of effect estimation for each medication was investigated using the surface under the surface under the cumulative ranking area (SUCRA) curve and mean ranks [17].

### Assessment of heterogeneity and inconsistency

We used the node-splitting method to assess the inconsistency of the model. When Bayesian *P*-value of model was less than 0.05, it was considered as the existence of significant inconsistency. We also used the Chi^2^ test and I^2^ statistics (heterogeneity variance parameter) to estimate the heterogeneity of the NMA, in which the heterogeneity between studies was defined as high if I^2^ > 50% and the random-effects model was used. On the contrary, the heterogeneity between studies was estimated as low and the fixed effects mode was used. We fitted the NMA model by calculating the ranking probabilities after the generation of heterogeneity matrix [7]. Moreover, the small-study effect was estimated by using funnel plots in this NMA [16].

### Sensitivity analysis and meta-regression

We planned a set of subgroup and sensitivity analyses to assess the effect of clinical and study design effect modifiers—e.g., MS, DS, DB, CD, SS, IS, ID, and RRB. The primary outcomes were separately analyzed for severe and non-severe COVID-19 patients as these patients might respond differently to pharmacological interventions.

During the treatment of COVID-19 patients, MS, DS, DB, CD, SS, IS, ID, and RRB might influence the data analysis of efficacy and safety. Thus, we investigated whether these covariates were related to change in COVID-19 parameters. We did meta-regressions aiming to examine the relationship between medication-associated COVID-19 therapy and MS, DS, DB, CD, SS, IS, ID, and RRB.

We used the netmeta package in R (version 4.0.4) to duplicate NMAs of the primary outcomes.

### Availability of data and materials

All relevant data to the study were included in the article or uploaded as supplementary information. Data is available upon reasonable request.

## RESULTS

### Characteristics and quality of included studies

**Figure 2:**
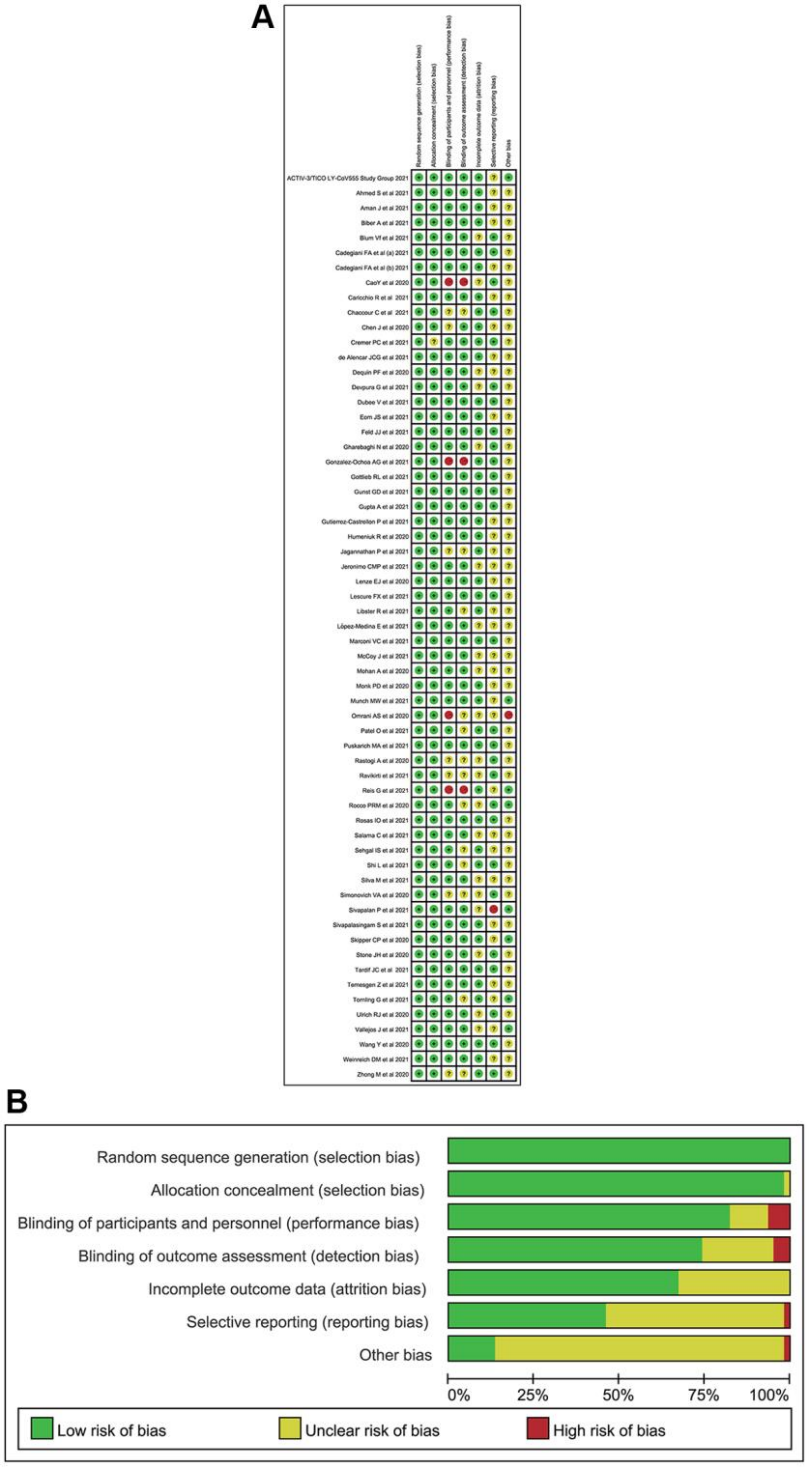
**The quality for included randomized placebo-controlled trials.** (**A**) Risk of bias summary (Note: The yellow circle with question mark represents “unclear risk of bias”, the red one with minus sign represents “high risk of bias” and the green one with plus sign represents “low risk of bias”). (**B**) Risk of bias graph.

We identified 3,869 citations through our searches, from which 73 RPCTs (57 in non-severe COVID-19 patients and 16 in severe COVID-19 patients), comprising 20,680 patients were selected. Sixty-one articles (i.e., 46 in non-severe COVID-19 [18–63], 15 in severe COVID-19 infection [64–78]) evaluating 49 different medications or placebo were included in this NMA, in which 12 articles were not yet published in peer-reviewed journals (Supplementary Table 1, Figure 1). Supplementary Table 1 summarized the characteristics of included studies. The mean sample size was 160 [interquartile range (IQR) 96–393] in this network analysis. The age of all patients was older than 18 years. The median duration of follow-up drugs intervention was 28 days (IQR 21–30). All 73 RPCTs performed the quality assessment using the Cochrane Risk-of-Bias Tool (Supplementary Tables 2 and 3). Overall, most of the studies were considered to be of good quality with low risk of bias (Figure 2, Supplementary Tables 2 and 3).

### Comparative efficacy and safety of pharmacological interventions

**Figure 3:**
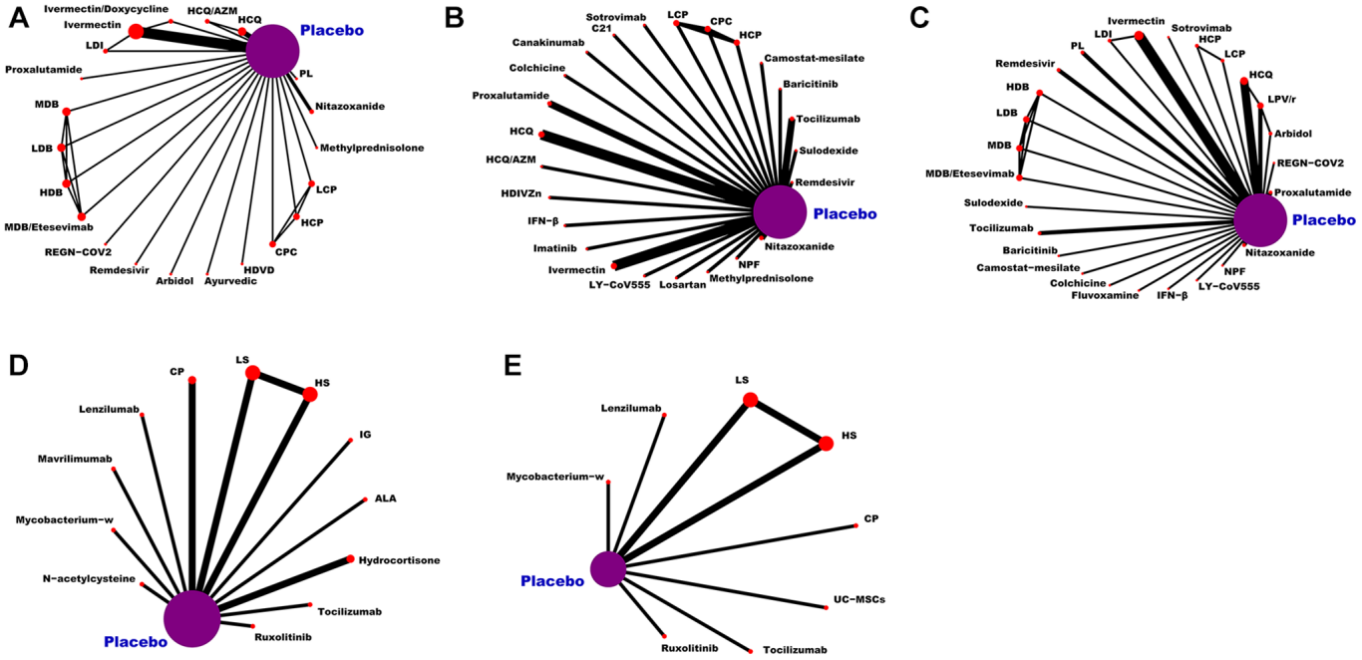
**Network plot of eligible comparisons for medications.** (**A**) The VC ratio of non-severe COVID-19 patients. (**B**) All-cause mortality of non-severe COVID-19 patients. (**C**) The TEAEs ratio of non-severe COVID-19 patients. (**D**) All-cause mortality of severe COVID-19 patients. (**E**) The TEAEs ratio of severe COVID-19 patients. Abbreviations: COVID-19: coronavirus disease 2019; VC: virological cure; TEAEs: treatment-emergent adverse events; PL: peginterferon lambda; LDI: low dosage ivermectin; LPV/r: lopinavir–ritonavir; AZM: azithromycin; HDVD: high-dose vitamin D; HDIVZn: high-dose intravenous zinc: LCP: low dosage CT-P59; HCP: high dosage CT-P59; CPC: CT-P59 combined; HCQ: hydroxychloroquine; LDB: low dosage bamlanivimab; MDB: moderate dosage bamlanivimab; HDB: high dosage bamlanivimab; LS: low dosage sarilumab; HS: high dosage sarilumab; NPF: novel probiotic formulation; CP: convalescent plasma; ALA: α-Lipoic acid; IFN-β: interferon beta; IG: immunoglobulin gamma.

As can be seen from Figure 3, the network of eligible comparisons for the efficacy and safety of pharmacological interventions. This NMA included 20,680 patients randomly assigned to 146 interventions or controls. In summary, this NMA presented well-connected nodes. All medications [e.g., α-Lipoic acid (ALA), hydroxychloroquine (HCQ), peginterferon lambda (PL), HCQ/azithromycin (HCQ/AZM), LY-CoV555, CP, remdesivir, proxalutamide, ivermectin/doxycycline (IDE), high-dose vitamin D (HDVD), canakinumab, camostat-mesilate, C21, ivermectin, colchicine, high-dose intravenous zinc (HDIVZn), interferon beta (IFN-β), LPV/r, low dosage CT-P59 (LCP), CT-P59 combined (CPC), high dosage CT-P59 (HCP), REGN-COV2, low dosage bamlanivimab (LDB), moderate dosage bamlanivimab (MDB), high dosage bamlanivimab (HDB), MDB/etesevimab, low dosage sarilumab (LS), high dosage sarilumab (HS), sotrovimab, sulodexide, novel probiotic formulation (NPF), losartan, ayurvedic, nitazoxanide, lenzilumab, hydrocortisone, imatinib, ruxolitinib, baricitinib, arbidol, fluvoxamine, immunoglobulin gamma (IG), low dosage ivermectin (LDI), mavrilimumab, methylprednisolone, mycobacterium-w, N-acetylcysteine, tocilizumab, and UC-MSCs] directly connected to placebo (Figure 3).

### The rate of virological cure for non-severe COVID-19 patients

**Figure 4:**
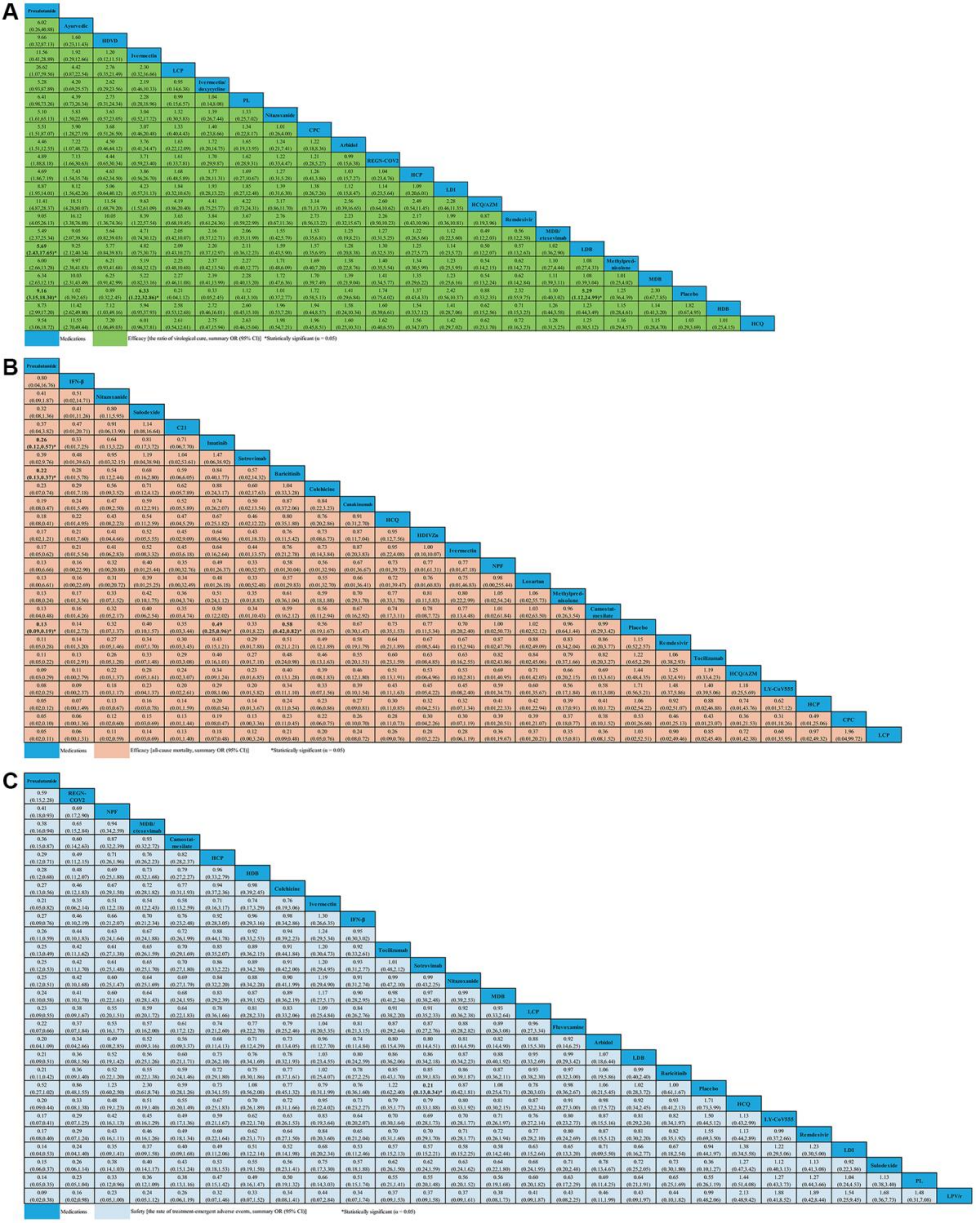
**Network meta-analyses of the relative efficacy and safety of medications in non-severe COVID-19 patients.** (**A**) The ratio of virological cure. (**B**) All-cause mortality. (**C**) The ratio of treatment-emergent adverse events. Abbreviations: COVID-19: coronavirus disease 2019; OR: odds ratio; CI: confidence interval; PL: peginterferon lambda; LDI: low dosage ivermectin; LPV/r: lopinavir–ritonavir; AZM: azithromycin; HDVD: high-dose vitamin D; HDIVZn: high-dose intravenous zinc; LCP: low dosage CT-P59; HCP: high dosage CT-P59; CPC: CT-P59 combined; HCQ: hydroxychloroquine; LDB: low dosage bamlanivimab; MDB: moderate dosage bamlanivimab; HDB: high dosage bamlanivimab; LS: low dosage sarilumab; HS: high dosage sarilumab; NPF: novel probiotic formulation; CP: convalescent plasma; ALA: α-Lipoic acid; IFN-β: interferon beta; IG: immunoglobulin gamma.

Twenty-one studies (*N* = 4,336), comprising of 29 RPCTs, contributed to the analysis of VC ratio (Supplementary Table 4). This NMA showed that 18 medications (e.g., arbidol, ayurvedic, CPC, HCP, HCQ, HCQ/AZM, HDB, HDVD, IDE, LCP, LDI, MDB, MDB/etesevimab, methylprednisolone, nitazoxanide, PL, REGN-COV2, and remdesivir) were not associated with an increased ratio of VC compared with placebo. Other medications, such as proxalutamide (OR 9.16, 95% CI 3.15–18.30), ivermectin (OR 6.33, 95% CI 1.22–32.86) and LDB (OR 5.29, 95% CI 1.12–24.99) seemed to significantly increase the ratio of VC compared with placebo (Figure 4A). Whilst the efficacy of proxalutamide for the VC was significantly better than LDB (OR 5.69, 95% CI 2.43–17.65) in patients with non-severe COVID-19 infection. However, no statistical difference for the VC of COVID-19 was found between proxalutamide and ivermectin (OR 11.56, 95% CI 0.41–28.89). The supplementary (Supplementary Figure 1) presented the ranking of the VC ratio for non-severe COVID-19 patients based on cumulative probability plots and SUCRA. The ranking for non-severe COVID-19 patients with the efficacy of VC ratio from high to low was as follows: proxalutamide (SUCRA: 92.9%), ayurvedic (SUCRA: 87.5%), HDVD (SUCRA: 79.8%), ivermectin (SUCRA: 77.6%), LCP (SUCRA: 60.0%), IDE (SUCRA: 59.8%), PL (SUCRA: 59.4%), nitazoxanide (SUCRA: 51.8%), CPC (SUCRA: 50.5%), arbidol (SUCRA: 48.8%), REGN-COV2 (SUCRA: 44.0%), HCP (SUCRA: 43.3%), LDI (SUCRA: 42.7%), HCQ/AZM (SUCRA: 41.1%), remdesivir (SUCRA: 39.3%), MDB/etesevimab (SUCRA: 35.7%), LDB (SUCRA: 35.1%), methylprednisolone (SUCRA: 33.5%), MDB (SUCRA: 32.4%), placebo (SUCRA: 31.1%), HDB (SUCRA: 30.1%), and HCQ (SUCRA: 28.4%) (Supplementary Figure 1).

### All-cause mortality for non-severe COVID-19 patients

For change in ACM, 29 studies compared 24 different medications (7,058 patients) with placebo (6,422 patients) in non-severe COVID-19 patients (Supplementary Table 5). We did not find evidence of ACM decreasing with C21, CPC, camostat-mesilate, canakinumab, colchicine, HCP, HCQ, HCQ/AZM, HDIVZn, IFN-β, ivermectin, LCP, LY-CoV555, losartan, methylprednisolone, NPF, nitazoxanide, remdesivir, sotrovimab, sulodexide, and tocilizumab when compared with placebo. We found evidence of ACM decreasing with proxalutamide (OR 0.13, 95% CI 0.09–0.19), imatinib (OR 0.49, 95% CI 0.25–0.96), and baricitinib (OR 0.58, 95% CI 0.42–0.82) (Figure 4B). Meanwhile, we found that proxalutamide seemed to be more effective than both imatinib (OR 0.26, 95% CI 0.12–0.57), and baricitinib (OR 0.22, 95% CI 0.13–0.37) for reducing the ACM of non-severe COVID-19 patients (Figure 4B). Based on cumulative probability plots and SUCRA, the supplementary (Supplementary Figure 2) presented the ranking for the ACM of medications in non-severe COVID-19 patients. The ranking for the ACM of non-severe COVID-19 patients from high to low was as follows: proxalutamide (SUCRA: 91.4%), IFN-β (SUCRA: 80.6%), nitazoxanide (SUCRA: 72.4%), sulodexide (SUCRA: 67.8%), C21 (SUCRA: 65.9%), imatinib (SUCRA: 64.7%), sotrovimab (SUCRA: 63.0%), baricitinib (SUCRA: 59.2%), colchicine (SUCRA: 58.0%), CPC (SUCRA: 53.9%), canakinumab (SUCRA: 52.4%), ivermectin (SUCRA: 49.9%), HCQ (SUCRA: 48.6%), HDIVZn (SUCRA: 45.8%), NPF (SUCRA: 43.3%), LCP (SUCRA: 42.3%), HCP (SUCRA: 41.4%), losartan (SUCRA: 41.4%), camostat-mesilate (SUCRA: 37.0%), methylprednisolone (SUCRA: 36.8%), placebo (SUCRA: 33.9%), remdesivir (SUCRA: 29.7%), tocilizumab (SUCRA: 26.9%), HCQ/AZM (SUCRA: 24.0%), and LY-CoV555 (SUCRA: 19.6%) (Supplementary Figure 2).

### The ratio of treatment-emergent adverse events for non-severe COVID-19 patients

For change in the ratio of TEAEs, 40 studies compared twenty-six different medications (7,857 patients) with placebo (6,681 patients) in non-severe COVID-19 patients (Supplementary Table 6). In terms of safety, only sotrovimab seemed to be associated with lower the ratio of TEAEs than placebo (OR 0.21, 95% CI 0.13–0.34). We analyzed other medications that were not statistically different from one another (Figure 4C). The supplementary (Supplementary Figure 3) presented the ranking for the TEAEs ratio of medications in non-severe COVID-19 patients according to cumulative probability plots and SUCRA. The ranking for non-severe COVID-19 patients with the ratio of TEAEs from high to low was as follows: proxalutamide (SUCRA: 98.7%), REGN-COV2 (SUCRA: 85.6%), NPF (SUCRA: 80.3%), MDB/etesevimab (SUCRA: 77.0%), camostat-mesilate (SUCRA: 73.2%), HCP (SUCRA: 62.3%), HDB (SUCRA: 59.9%), colchicine (SUCRA: 59.1%), ivermectin (SUCRA: 58.7%), IFN-β (SUCRA: 56.5%), sotrovimab (SUCRA: 53.1%), tocilizumab (SUCRA: 52.8%), nitazoxanide (SUCRA: 52.2%), MDB (SUCRA: 50.6%), LCP (SUCRA: 45.9%), fluvoxamine (SUCRA: 44.5%), arbidol (SUCRA: 43.2%), LDB (SUCRA: 42.1%), baricitinib (SUCRA: 40.1%), placebo (SUCRA: 39.5%), HCQ (SUCRA: 36.6%), LY-CoV555 (SUCRA: 29.2%), remdesivir (SUCRA: 29.2%), LDI (SUCRA: 25.0%), sulodexide (SUCRA: 23.8%), PL (SUCRA: 18.8%), and LPV/r (SUCRA: 11.9%) (Supplementary Figure 3).

### All-cause mortality for severe COVID-19 patients

**Figure 5:**
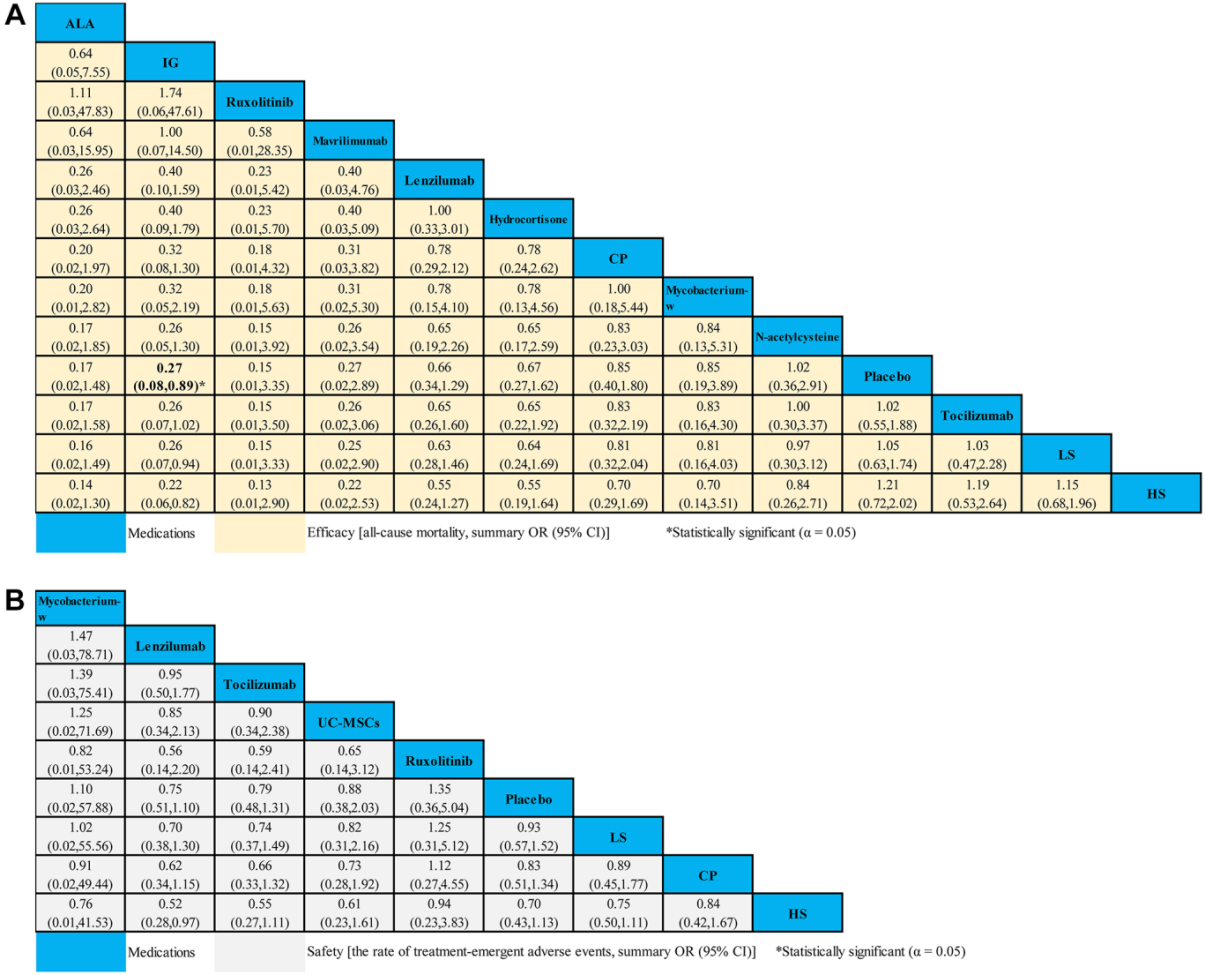
**Network meta-analyses of the relative efficacy and safety of medications in severe COVID-19 patients.** (**A**) All-cause mortality. (**B**) The ratio of treatment-emergent adverse events. Abbreviations: COVID-19: coronavirus disease 2019; OR: odds ratio; CI: confidence interval; LS: low dosage sarilumab; HS: high dosage sarilumab; CP: convalescent plasma; ALA: α-Lipoic acid; IG: immunoglobulin gamma.

Fourteen studies compared 16 different medications (2,008 patients) with placebo (1,081 patients) contributed to this analysis on ACM in severe COVID-19 patients (Supplementary Table 7). Compared with placebo, we only found that IG (OR 0.27, 95% CI 0.08–0.89) seemed to be associated with decreased ACM (Figure 5A). However, no statistical difference for the ACM in severe COVID-19 patients was found in other medications (Figure 5A). Based on cumulative probability plots and SUCRA, the supplementary (Supplementary Figure 4) presented the ranking for the ACM of medications in severe COVID-19 patients. The ranking for the ACM of severe COVID-19 patients from high to low was as follows: ALA (SUCRA: 83.0%), IG (SUCRA: 80.7%), ruxolitinib (SUCRA: 79.8%), mavrilimumab (SUCRA: 72.6%), lenzilumab (SUCRA: 55.3%), hydrocortisone (SUCRA: 53.8%), CP (SUCRA: 41.6%), mycobacterium-w (SUCRA: 40.8%), N-acetylcysteine (SUCRA: 32.4%), placebo (SUCRA: 31.3%), tocilizumab (SUCRA: 30.8%), LS (SUCRA: 28.6%), and HS (SUCRA: 19.1%) (Supplementary Figure 4).

### The ratio of treatment-emergent adverse events for severe COVID-19 patients

A total of 8 studies compared eight different medications (1,316 patients) with placebo (692 patients) formed the evidence network for the TEAEs ratio in severe COVID-19 patients (Supplementary Table 8). Compared with placebo, we found no strong evidence of change in the TEAEs ratio with 8 medications for severe COVID-19 patients (Figure 5B). According to cumulative probability plots and SUCRA, the supplementary (Supplementary Figure 5) presented the ranking for the TEAEs ratio of medications in severe COVID-19 patients. The ranking for non-severe COVID-19 patients with the ratio of TEAEs from high to low was as follows: mycobacterium-w (SUCRA: 80.6%), lenzilumab (SUCRA: 66.1%), tocilizumab (SUCRA: 60.1%), UC-MSCs (SUCRA: 53.5%), ruxolitinib (SUCRA: 53.3%), placebo (SUCRA: 51.3%), LS (SUCRA: 35.7%), CP (SUCRA: 31.3%), and HS (SUCRA: 30.5%) (Supplementary Figure 5).

### Evaluation of inconsistency

As shown in Table 1, based on the Chi² and *P* values, we did not find a significant inconsistency for the efficacy and safety outcomes in severe or non-severe patients with COVID-19 infection. In terms of node-splitting, statistical significance was not shown as the local tests of loop inconsistency (Supplementary Table 9).

**Table 1:** The evaluation of inconsistency for the efficacy and safety of medications.

**Network meta-analysis**	**Number of dimensions**	**Chi^2^ value**	***P* value**
**Non-severe COVID-19 patients**			
The ratio of virological cure	21	3.46	0.326
All-cause mortality	24	0.98	0.996
The ratio of treatment-emergent adverse events	26	1.56	0.668
**Severe COVID-19 patients**			
All-cause mortality	12	0.02	1.000
The ratio of treatment-emergent adverse events	8	0.04	1.000

Abbreviation: COVID-19: coronavirus disease 2019.

### Assessment of small study effects

In general, there was no evidence of small study effects for NMAs based on funnel plot symmetry (Supplementary Figure 6). Direct and indirect evidence showed high agreement throughout NMAs, thus meeting the condition of consistency.

### Sensitivity analyses

**Figure 6:**
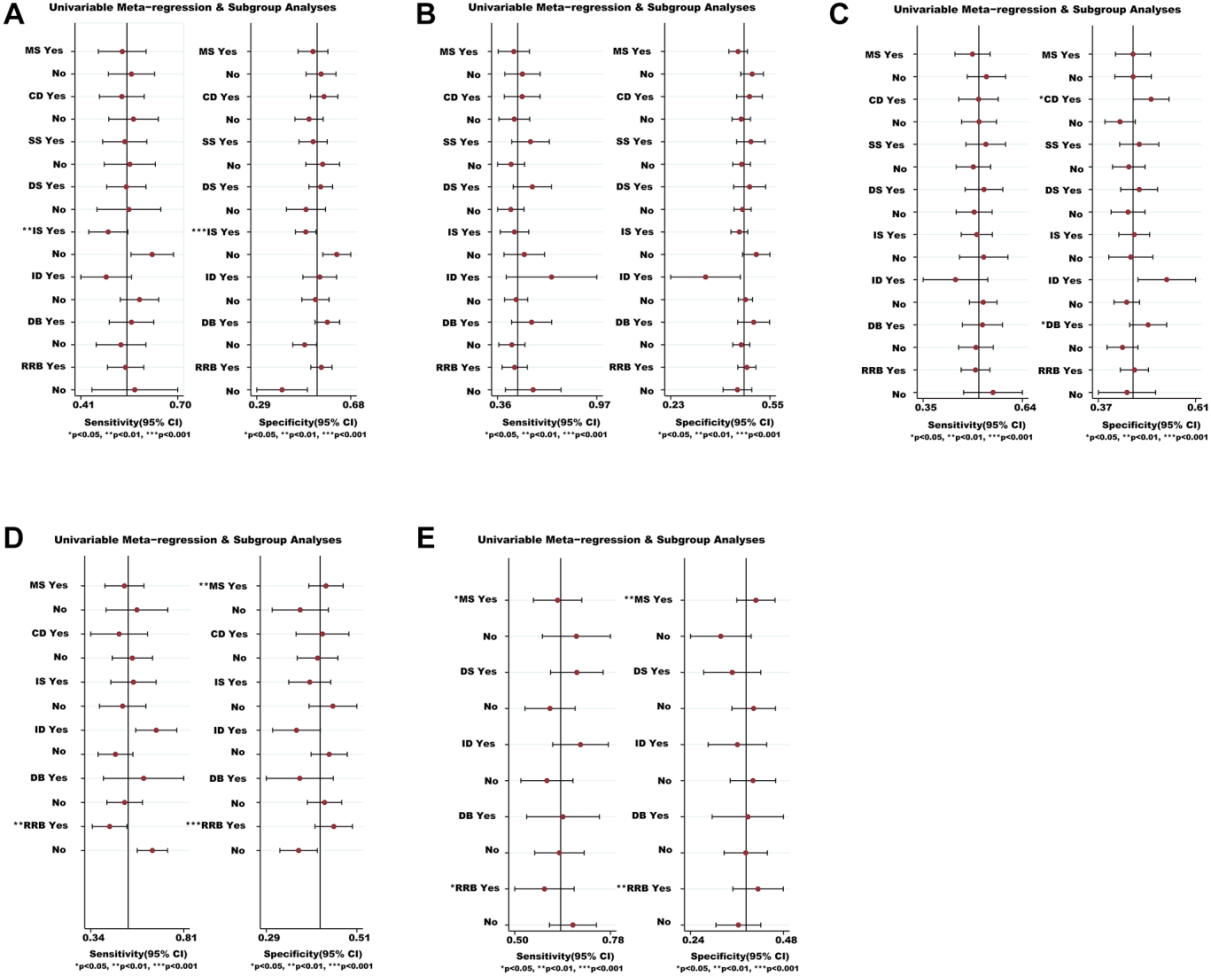
**Meta-regression and sensitivity analyses for the efficacy and safety of medications.** (**A**) The VC ratio of non-severe COVID-19 patients. (**B**) All-cause mortality of non-severe COVID-19 patients. (**C**) The TEAEs ratio of non-severe COVID-19 patients. (**D**) All-cause mortality of severe COVID-19 patients. (**E**) The TEAEs ratio of severe COVID-19 patients. ^*^We conducted meta-regression and sensitivity analyses to estimate the impact of variable for each outcome. The potential modifiers (variables) for meta-regression we select are listed below: MS, DS, DB, CD, SS, IS, ID, and RRB. Abbreviations: COVID-19: coronavirus disease 2019; VC: virological cure; TEAEs: treatment-emergent adverse events; MS: multicenter study; DS: duration of study; DB: double blind; CD: crossover design; SS: sample size; IS: industry sponsorship; ID: inequalities in doses; RRB: risk of reported bias.

We analyzed the possible sources of heterogeneity or inconsistency by using subgroup and meta-regression analyses. Sensitivity analyses showed that most modifiers (such as CD, SS, DS, ID and DB) did not significantly affect the efficacy and safety of medications (Figure 6). However, we found that there was a significant heterogeneity source (i.e., IS) for the VC (*P* < 0.01) in non-severe COVID-19 patients (Figure 6A). We also found that the RRB was the heterogeneity source of ACM for severe COVID-19 patients based on sensitivity analysis (*P* < 0.01) (Figure 6D). Whilst the MS and RRB seemed to be associated with the TEAEs ratio of severe COVID-19 patients (*P* < 0.05) (Figure 6E).

Finally, we duplicated NMAs of the primary outcomes, which was consistent with the present findings using the netmeta package of R software.

## DISCUSSION

Previous studies might be biased because pharmacological interventions or the controls were not standardized before performing a meta-analysis [8–10, 79]. To address this shortfall, we performed an updated NMA study based on RPCTs. This updated NMA is based on the studies of 73 RPCTs, which included 20,680 patients randomly assigned to 49 different drug interventions or placebo. The present NMA is essentially more acceptable because it only included the RPCTs (i.e., standardizing the controls), and analyzed the data based on the stratification of COVID-19 infection status (such as severe and non-severe patients).

### Efficacy of current medications in severe or non-severe patients with COVID-19

We found that medications vary markedly in their efficacy and safety between severe and non-severe COVID-19 infection. Of all the included active interventions, only proxalutamide, ivermectin, and LDB were significantly more efficacious than placebo, in which the efficacy of proxalutamide seemed to be better for the VC in non-severe COVID-19 patients. Prior studies have shown that proxalutamide was effective in eradicating SARS coronavirus [80]. As expected, this finding further supported the work of other studies in this area linking proxalutamide with the viral eradication of SARS-Coronavirus-2. Unfortunately, we were unable to get the relevant data of VC for patients with severe COVID-19 infection in this study. The reason for this is unclear, but maybe researchers think it is not a primary outcome in severe COVID-19 patients. For change in ACM, we found that proxalutamide, imatinib, and baricitinib were more efficacious than placebo for patients with non-severe COVID-19 infection; however, we only found that IG was associated with decreased ACM in severe COVID-19 patients. Interestingly, for decreasing ACM, we also found that proxalutamide was better than other medications among patients with non-severe COVID-19. Proxalutamide seemed to be the best choice based on efficacy in non-severe COVID-19 patients [23, 24].

We found that LDB seemed to be associated with the VC of non-severe COVID-19 patients. However, for higher doses of bamlanivimab we did not find evidence of an effect versus placebo in terms of VC. It seemed that we could not observe bamlanivimab with an apparent dose–response relationship from the VC of non-severe COVID-19 infection. There are several possible explanations for this considerable difference. First, bamlanivimab with higher doses might be detrimental to innate immune regulation and VC [81]. Second, those higher doses of bamlanivimab might unfavorably change the balance between regulatory T cells and Th17 cells [82–84]. Consistent with the literature [85], this research found that ivermectin was effective in patients with non-severe COVID-19. Thus, we suggested that ivermectin might be a better choice for the treatment of non-severe COVID-19 infection.

Further, our NMA identified that IG, proxalutamide, baricitinib, and imatinib were beneficial to the outcome of COVID-19 infection based on ACM. We suggested that IG might be a choice in the treatment of severe COVID-19 patients, and proxalutamide, baricitinib, and imatinib should be used for the therapy of non-severe COVID-19 patients. If so, perhaps patients and clinicians should carefully balance the risk-benefit profile to select drug interventions based on efficacy and safety between severe and non-severe patients with COVID-19. However, the interpretation of this result might be limited by insufficient medications selection (i.e., so far, the limited evidence for the therapy of COVID-19 patients) [4].

Undeniably, we should interpret cautiously these findings due to heterogeneity sources including IS and RRB (Figure 6). To verify the value of medications, we need to wait for large-scale RPCTs with target population, sensitive endpoints, and standardized study design among COVID-19 patients. Although previous studies have verified that steroid and auxora, and so on [86, 87] were efficacious in clinical practice, we did not include studies of these medications due to the inclusion criterion of RPCTs. Additionally, to identify our findings, further studies need to be conducted by using a stratified analysis based on the reference of different controls.

### Safety of current medications in severe or non-severe patients with COVID-19

As shown in Figures 4 and 5, we only found that sotrovimab was associated with a decrease in TEAEs for non-severe COVID-19 patients when compared with placebo; however, for all medications we did not find evidence of a safety versus placebo in terms of TEAEs among severe COVID-19 patients. Curiously, previous studies have indicated that some of the drugs were shown to perform better than placebo on safety: for instance, when compared with placebo, CP, tocilizumab, and ruxolitinib led to reductions in TEAEs in severe COVID-19 patients [88–90]. A possible explanation for this was that we did not include sufficient data in this network analysis. Of note, according to the result of sensitivity analysis, the present finding may need further verification. Therefore, statistical indications of clinical superiority in this study required careful interpretation.

### Limitations

Our analysis had some limitations. First, despite attempts made to include all available RPCTs, we were not able to exclude the possibility of missing data. Second, we only extracted three types of endpoints in the published data. We did not analyze other important outcomes (e.g., discharge ratio and intensive care unit admission). Although we tried to collect better indicators, most studies did not indicate the definition of biological and clinical outcomes. Third, we analyzed only pooled treatment effects and were unable to investigate potentially important clinical and demographic modifiers of treatment response at the individual patient level (i.e., age, sex, severity of symptoms, and duration of illness). Fourth, this NMA did not include unpublished data. Additionally, some nodes in our NMA included only a few trials. The sample size of the actual head-to-head RPCTs was small. Hence, we frequently analyzed their efficacy and safety in different drug interventions through indirect comparisons. Fifth, our study only acute efficacy/adverse events were examined and that more data on potential long-term effects were needed. Moreover, the CIs of effect size estimates were relatively wide, which might affect the reliability of our findings in this NMA. Finally, we found the statistical heterogeneousness in this NMA based on sensitivity analyses. For example, IS, MS, and RRB might conceal or exaggerate the effect size of this NMA. Further research should be undertaken to control these confounding factors.

## CONCLUSIONS

In conclusion, marked variations exist in the efficacy and safety of medications between severe and non-severe patients with COVID-19. Compared with placebo, of all the included active interventions, only proxalutamide, ivermectin, and LDB might be more efficacious than placebo for the VC ratio in non-severe COVID-19 patients; however, we were not able to get the relevant data of VC for severe COVID-19 patients in this NMA. We found that proxalutamide, imatinib, and baricitinib might be associated with the decrease of ACM among non-severe COVID-19 patients; however, for decrease in ACM, we only verified that IG might be related to severe COVID-19 infection. Among them, proxalutamide seemed to be a good choice for the therapy of COVID-19. Based on safety, we suggested that sotrovimab might benefit the treatment of non-severe COVID-19 patients; however, for change in TEAEs, the difference was not found in all included medications from severe COVID-19 patients.

Notwithstanding these limitations, the findings from this NMA may represent a more comprehensive analysis of the available evidence. It seems that monoclonal antibodies (e.g., LDB, baricitinib, imatinib, and sotrovimab) are a better choice for treating severe or non-severe COVID-19 patients. However, clinical decisions to use preferentially medications should carefully consider the risk-benefit profile based on efficacy and safety of all active interventions in patients with COVID-19 at different levels of infection. Treatment guidelines should be updated to reflect differences in the degree of infection, but the selection of the treatment intervention should be made on a case-by-case basis, considering the clinical circumstances and preferences of patients and clinicians. We hope that these findings will assist in shared decision making between patients and their clinicians. To be sure, more large-scale RPCTs and big data analysis should be collaborated and performed for the treatment of COVID-19 infection. Thus, the prevention and therapy of COVID-19 is set to change for better in the future.

## Supplementary Materials

Supplementary Figures

Supplementary Table 1

Supplementary Table 2

Supplementary Tables 3-9

Appendix 1

Appendix 2
