# Supplementary material for: Efficacy and safety of current medications for treating severe and non-severe COVID-19 patients: an updated network meta-analysis of randomized placebo-controlled trials
Source: Aging (Albany NY). 2021 Sep 16;13(18):21866–902. doi: 10.18632/aging.203522 (PMC8507270; doi:10.18632/aging.203522)
Supplement: Supplementary Figures [file aging-13-203522-s001.pdf]

SUPPLEMENTARY FIGURES

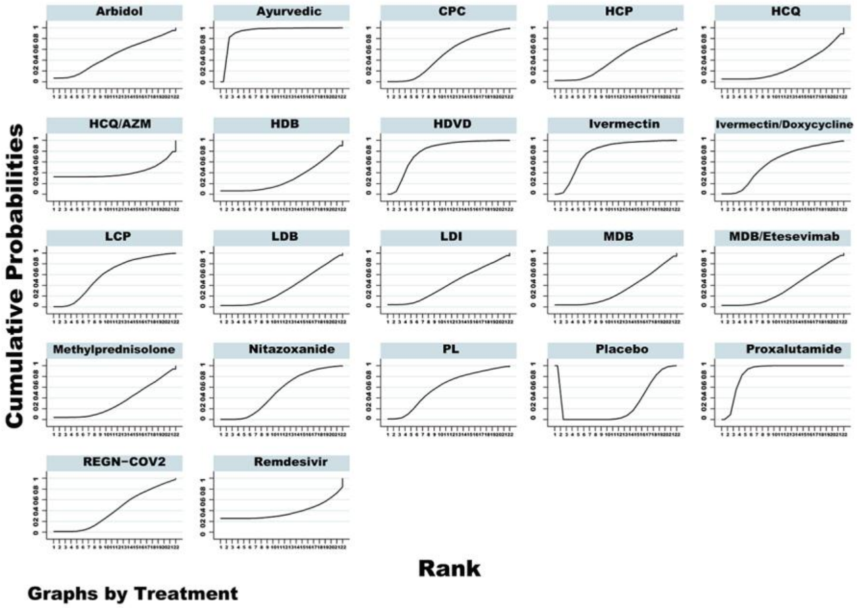

Treatment Relative Ranking of Model 1

Treatment	SUCRA	PrBest	MeanRank
Placebo	31.1	0.0	15.5
Arbidol	48.8	6.7	11.7
Ayurvedic	92.9	0.0	2.5
CPC	50.5	0.3	11.4
HCP	43.3	2.4	12.9
HCQ	28.4	4.8	16.0
HCQ/AZM	41.1	32.4	13.4
HDB	30.1	6.5	15.7
HDVD	79.8	0.0	5.3
Ivermectin	77.6	0.1	5.7
Ivermectin/Doxycycline	59.8	0.8	9.5
LCP	60.0	0.5	9.4
LDB	35.1	2.6	14.6
LDI	42.7	4.3	13.0
MDB	32.4	3.7	15.2
MDB/Etesevimab	35.7	2.7	14.5
Methylprednisolone	33.5	4.0	15.0
Nitazoxanide	51.8	0.3	11.1
PL	59.4	1.0	9.5
Proxalutamide	87.5	0.0	3.6
REGN-COV2	44.0	1.3	12.8
Remdesivir	39.3	25.5	13.7

Supplementary Figure 1. Treatment ranking and SUCRA plot for the virological cure ratio among non-severe COVID-19 patients. *Larger SUCRAs denote more effective interventions. Abbreviations: SUCRA: surface under the cumulative ranking area; COVID-19: coronavirus disease 2019; PL: peginterferon lambda; LDI: low dosage ivermectin; AZM: azithromycin; HDVD: high-dose vitamin D; LCP: low dosage CT-P59; HCP: high dosage CT-P59; CPC: CT-P59 combined; HCQ: hydroxychloroquine; LDB: low dosage bamlanivimab; MDB: moderate dosage bamlanivimab; HDB: high dosage bamlanivimab.

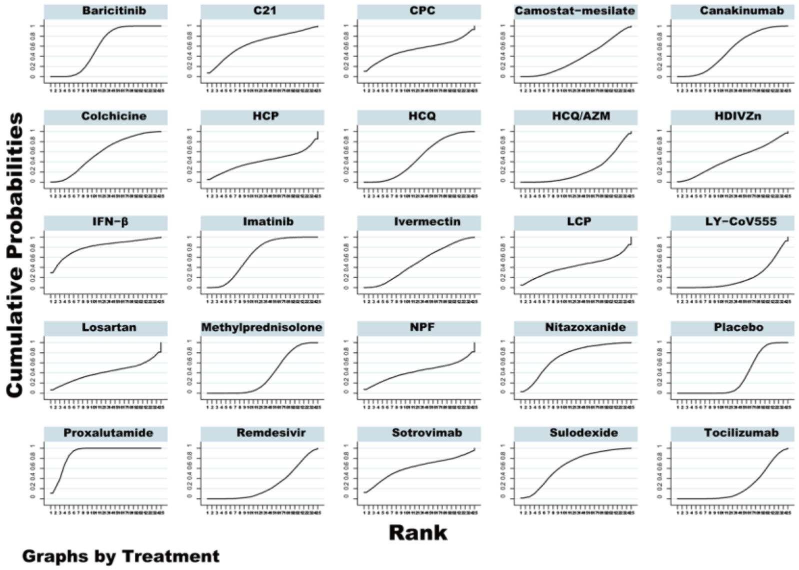

Treatment Relative Ranking of Model 1

Treatment	SUCRA	PrBest	MeanRank
Placebo	33.9	0.0	16.9
Baricitinib	59.2	0.0	10.8
C21	65.9	7.1	9.2
CPC	53.9	10.5	12.1
Camostat-mesilate	37.0	0.0	16.1
Canakinumab	52.4	0.0	12.4
Colchicine	58.0	0.1	11.1
HCP	41.4	4.9	15.1
HCQ	48.6	0.0	13.3
HCQ/AZM	24.0	0.0	19.2
HDIVZn	45.8	0.7	14.0
IFN-β	80.6	29.7	5.6
Imatinib	64.7	0.0	9.5
Ivermectin	49.9	0.1	13.0
LCP	42.3	5.1	14.8
LY-CoV555	19.6	0.0	20.3
Losartan	41.4	6.6	15.1
Methylprednisolone	36.8	0.0	16.2
NPF	43.3	7.5	14.6
Nitazoxanide	72.4	2.9	7.6
Proxalutamide	91.4	11.1	3.1
Remdesivir	29.7	0.0	17.9
Sotrovimab	63.0	12.5	9.9
Sulodexide	67.8	1.2	8.7
Tocilizumab	26.9	0.0	18.5

Supplementary Figure 2. Treatment ranking and SUCRA plot for all-cause mortality among non-severe COVID-19 patients. *Larger SUCRAs denote less all-cause mortality. Abbreviations: SUCRA: surface under the cumulative ranking area; COVID-19: coronavirus disease 2019; HCQ: hydroxychloroquine; AZM: azithromycin; HDIVZn: high-dose intravenous zinc; IFN-β: interferon beta; NPF: novel probiotic formulation; LCP: low dosage CT-P59; HCP: high dosage CT-P59; CPC: CT-P59 combined.

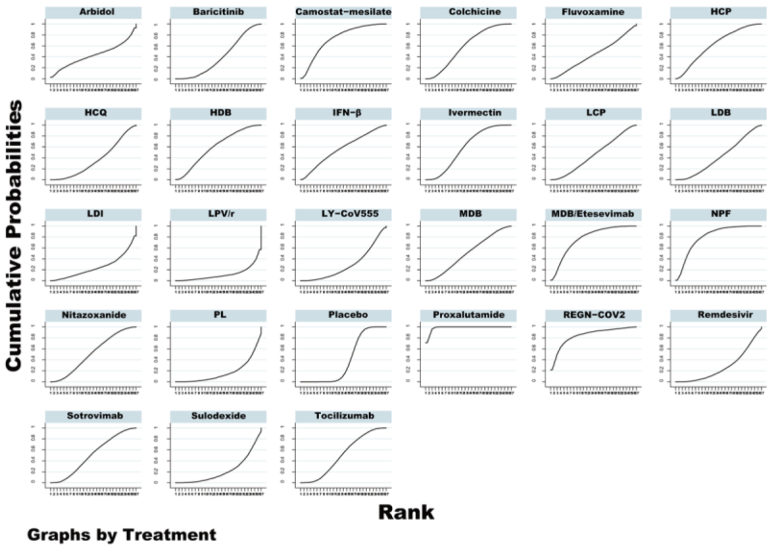

Treatment Relative Ranking of Model 1

Treatment	SUCRA	PrBest	MeanRank
Placebo	39.5	0.0	16.7
Arbidol	43.2	3.0	15.8
Baricitinib	40.1	0.0	16.6
Camostat-mesilate	73.2	0.7	8.0
Colchicine	59.1	0.0	11.6
Fluvoxamine	44.5	0.2	15.4
HCP	62.3	0.3	10.8
HCQ	36.6	0.0	17.5
HDB	59.9	0.1	11.4
IFN-β	56.5	0.3	12.3
Ivermectin	58.7	0.0	11.7
LCP	45.9	0.0	15.1
LDB	42.1	0.0	16.0
LDI	25.0	0.1	20.5
LPV/r	11.9	0.0	23.9
LY-CoV555	29.2	0.0	19.4
MDB	50.6	0.0	13.8
MDB/Etesevimab	77.0	1.2	7.0
NPF	80.3	0.9	6.1
Nitazoxanide	52.2	0.0	13.4
PL	18.8	0.0	22.1
Proxalutamide	98.7	71.4	1.3
REGN-COV2	85.6	21.7	4.7
Remdesivir	29.2	0.0	19.4
Sotrovimab	53.1	0.0	13.2
Sulodexide	23.8	0.0	20.8
Tocilizumab	52.8	0.0	13.3

Supplementary Figure 3. Treatment ranking and SUCRA plot for the ratio of treatment-emergent adverse events among non-severe COVID-19 patients. *Larger SUCRAs denote less treatment-emergent adverse events. Abbreviations: SUCRA: surface under the cumulative ranking area; COVID-19: coronavirus disease 2019; PL: peginterferon lambda; LDI: low dosage ivermectin; IFN-β: interferon beta; NPF: novel probiotic formulation; LPV/r: lopinavir–ritonavir; LCP: low dosage CT-P59; HCP: high dosage CT-P59; LDB: low dosage bamlanivimab; MDB: moderate dosage bamlanivimab; HDB: high dosage bamlanivimab.

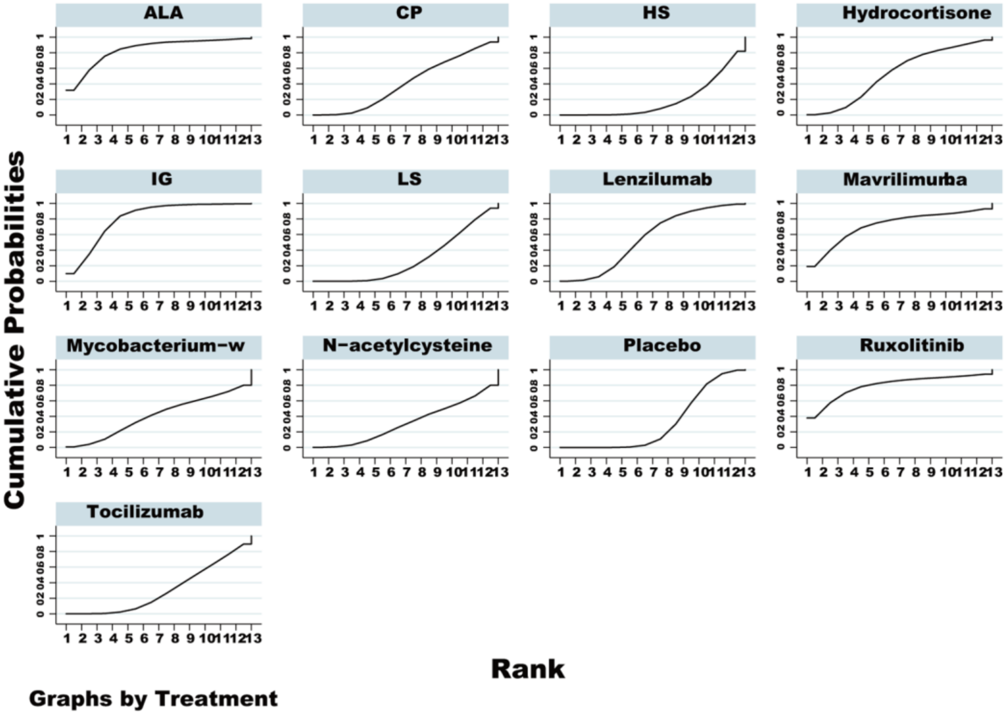

Treatment Relative Ranking of Model 1

Treatment	SUCRA	PrBest	MeanRank
Placebo	31.3	0.0	9.2
ALA	83.0	29.2	3.0
CP	41.6	0.0	8.0
HS	19.1	0.0	10.7
Hydrocortisone	53.8	0.2	6.5
IG	80.7	9.2	3.3
LS	28.6	0.0	9.6
Lenzilumab	55.3	0.2	6.4
Mavrilimumab	72.6	19.6	4.3
Mycobacterium-w	40.8	0.8	8.1
N-acetylcysteine	32.4	0.1	9.1
Ruxolitinib	79.8	40.6	3.4
Tocilizumab	30.8	0.0	9.3

Supplementary Figure 4. Treatment ranking and SUCRA plot for all-cause mortality among severe COVID-19 patients. *Larger SUCRAs denote less treatment-emergent adverse events. Abbreviations: SUCRA: surface under the cumulative ranking area; COVID-19: coronavirus disease 2019; LS: low dosage sarilumab; HS: high dosage sarilumab; CP: convalescent plasma; ALA: α-Lipoic acid; IG: immunoglobulin gamma.

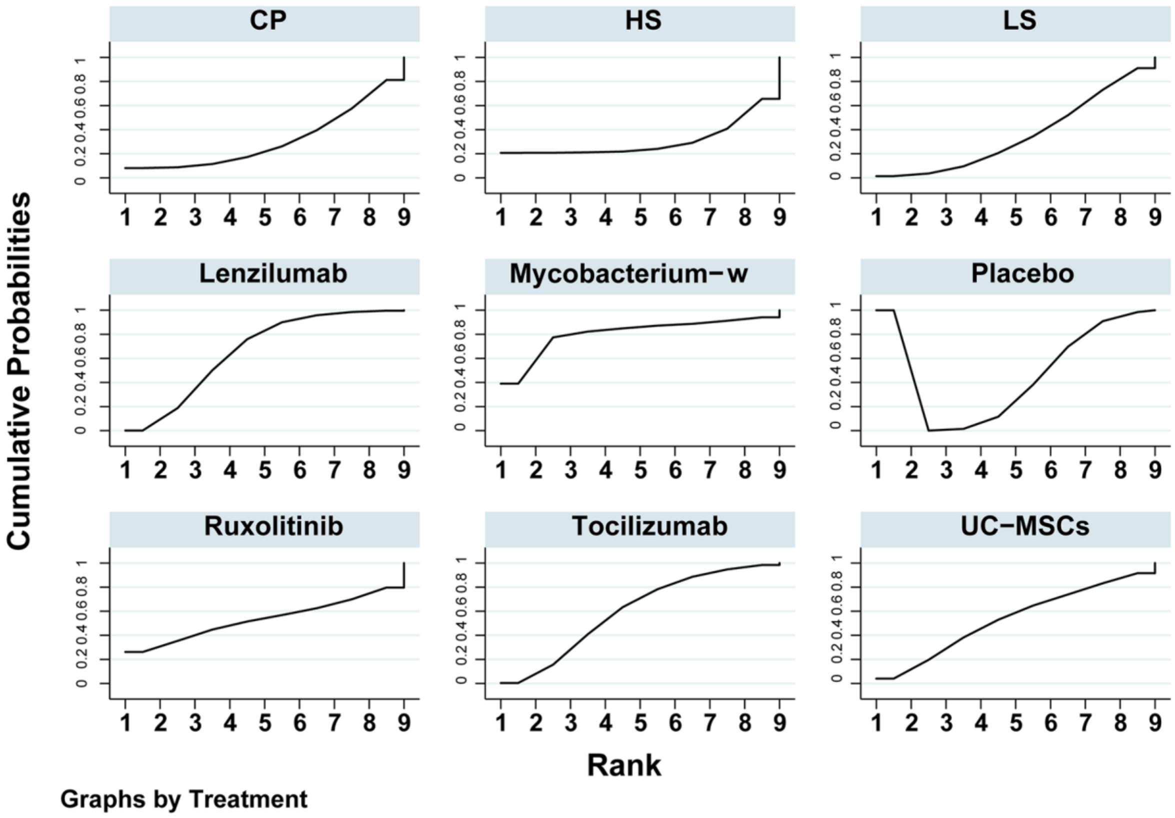

Treatment Relative Ranking of Model 1

Treatment	SUCRA	PrBest	MeanRank
Placebo	51.3	0.1	4.9
CP	31.3	8.1	6.5
HS	30.5	20.7	6.6
LS	35.7	1.4	6.1
Lenzilumab	66.1	0.1	3.7
Mycobacterium-w	80.6	39.0	2.6
Ruxolitinib	53.3	26.2	4.7
Tocilizumab	60.1	0.4	4.2
UC-MSCs	53.5	4.0	4.7

Supplementary Figure 5. Treatment ranking and SUCRA plot for the ratio of treatment-emergent adverse events among severe COVID-19 patients. Severe COVID-19 patients. *Larger SUCRAs denote less treatment-emergent adverse events. Abbreviations: SUCRA: surface under the cumulative ranking area; COVID-19: coronavirus disease 2019; LS: low dosage sarilumab; HS: high dosage sarilumab; CP: convalescent plasma.

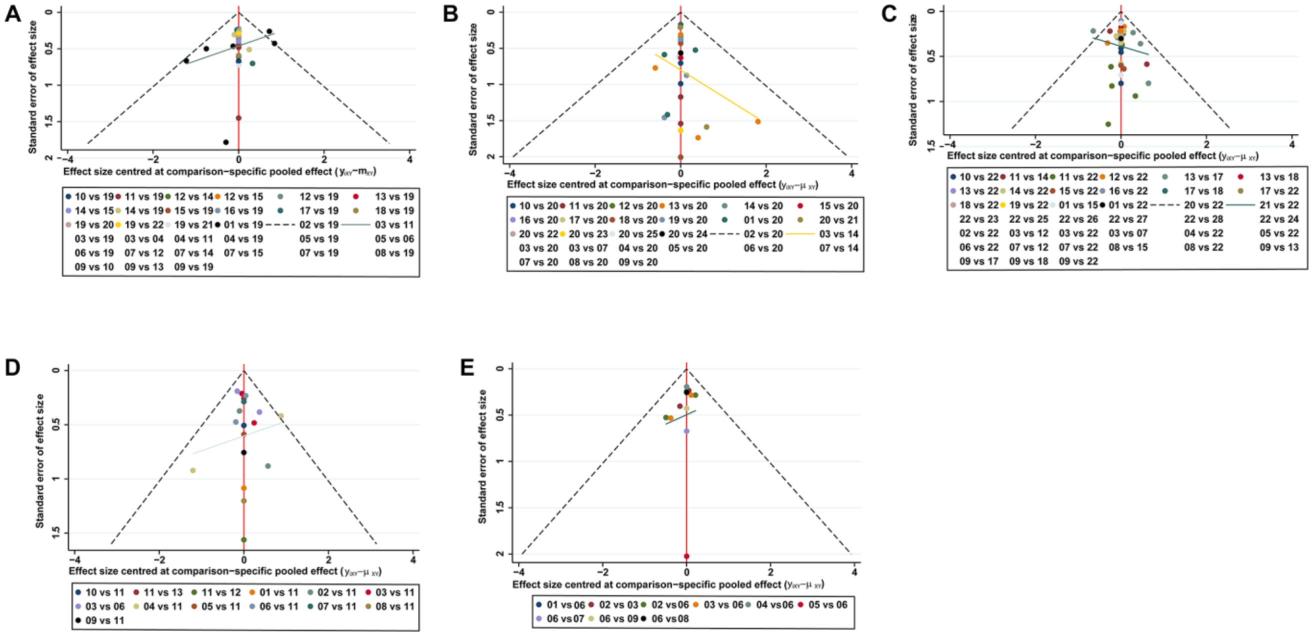

Supplementary Figure 6. Funnel plots of publication bias for the efficacy and safety of medications. (**A**) the ratio of virological cure for non-severe COVID-19 patients (01: arbidol; 02: ayurvedic; 03: CPC; 04: HCP; 05: HCQ; 06: HCQ/AZM; 07: HDB; 08: HDVD; 09: ivermectin; 10: ivermectin/doxycycline; 11: LCP; 12: LDB; 13: LDI; 14: MDB; 15: MDB/etesevimab; 16: methylprednisolone; 17: nitazoxanide; 18: PL; 19: placebo; 20: proxalutamide; 21: REGN-COV2; 22: remdesivir). (**B**) All-cause mortality for non-severe COVID-19 patients (01: baricitinib; 02: C21; 03: CPC; 04: camostat-mesilate; 05: canakinumab; 06: colchicine; 07: HCP; 08: HCQ; 09: HCQ/AZM; 10: HDIVZn; 11: IFN-β; 12: imatinib; 13: ivermectin; 14: LCP; 15: LY-CoV555; 16: losartan; 17: methylprednisolone; 18: NPF; 19: nitazoxanide; 20: placebo; 21: proxalutamide; 22: remdesivir; 23: sotrovimab; 24: sulodexide; 25: tocilizumab). (**C**) The ratio of treatment-emergent adverse events for non-severe COVID-19 patients (01: arbidol; 02: baricitinib; 03: camostat-mesilate; 04: colchicine; 05: fluvoxamine; 06: HCP; 07: HCQ; 08: HDB; 09: IFN-β; 10: ivermectin; 11: LCP; 12: LDB;13: LDI; 14: LPV/r; 15: LY-CoV555; 16: MDB; 17: MDB/etesevimab; 18: NPF; 19: nitazoxanide; 20: PL; 21: placebo; 22: proxalutamide; 23: REGN-COV2; 24: remdesivir; 25: sotrovimab; 26: sulodexide; 27: tocilizumab). (**D**) All-cause mortality of severe COVID-19 patients (01: ALA; 02: CP; 03: HS; 04: hydrocortisone; 05: IG; 06: LS; 07: lenzilumab; 08: mavrilimumab; 09: mycobacterium-w; 10: N-acetylcysteine; 11: placebo; 12: ruxolitinib; 13: tocilizumab). (**E**). The TEAEs ratio of severe COVID-19 patients (01: CP; 02: HS; 03: LS; 04: lenzilumab; 05: mycobacterium-w; 06: placebo; 07: ruxolitinib; 08: tocilizumab; 09: UC-MSCs). Abbreviations: COVID-19: coronavirus disease 2019; VC: virological cure; TEAEs: treatment-emergent adverse events; PL: peginterferon lambda; LDI: low dosage ivermectin; LPV/r: lopinavir–ritonavir; AZM: azithromycin; HDVD: high-dose vitamin D; HDIVZn: high-dose intravenous zinc: LCP: low dosage CT-P59; HCP: high dosage CT-P59; CPC: CT-P59 combined; HCQ: hydroxychloroquine; LDB: low dosage bamlanivimab; MDB: moderate dosage bamlanivimab; HDB: high dosage bamlanivimab; LS: low dosage sarilumab; HS: high dosage sarilumab; NPF: novel probiotic formulation; CP: convalescent plasma; ALA: α-Lipoic acid; IFN-β: interferon beta; IG: immunoglobulin gamma.
