# Supplementary material for: Efficacy and safety of current medications for treating severe and non-severe COVID-19 patients: an updated network meta-analysis of randomized placebo-controlled trials
Source: Aging (Albany NY). 2021 Sep 16;13(18):21866–902. doi: 10.18632/aging.203522 (PMC8507270; doi:10.18632/aging.203522)
Supplement: Supplementary Table 1 [file aging-13-203522-s002.docx]

| **Supplementary Table 1. Characteristics of randomized placebo-controlled trials of pharmacological interventions versus placebo in severe or non-severe patients with COVID-19.** | | | | | | | | | | | | | | |
| --- | --- | --- | --- | --- | --- | --- | --- | --- | --- | --- | --- | --- | --- | --- |
| **Author name (reference)** | **Publication year** | **Country/ Countries of origin** | **Study design** | **Method of COVID-19**  **testing** | **Patient population** | **Numbers of participants** | **Gender*** | **Age (years)** | **Interventions** | **Treatment medication dose** | **Controls** | **Control medication dose** | **Follow- up time (days)** | **Primary outcomes** |
| ACTIV-3/TICO LY-  CoV555 Study Group [18] | 2021 | USA,  Denmark, Singapore | MDRPCTs | RT-PCR | Non-severe and hospitalized patients | 314 | Males (n =177);  Females (n =137) | ≥ 18 | LY-CoV555 (n =163) | LY-CoV555 (at a dose of 7000 mg), a neutralizing monoclonal antibody | Placebo (n= 151) | Not specified | 90 | ACM; ratio of TEAEs |
| Ahmed S et al [19] | 2021 | Bangladesh | DRPCTs | RT-PCR | Non-severe and hospitalized patients | 72 | Not mentioned | 18 to 65 | Ivermectin (n = 24) | Oral ivermectin alone (12 mg once daily for 5 days) | Placebo (n  = 24) | Not specified | 14 | Ratio of VC |
| Ahmed S et al [19] | 2021 | Bangladesh | DRPCTs | RT-PCR | Non-severe and hospitalized patients | 72 | Not mentioned | 18 to 65 | Ivermectin/Doxycycline (n  = 24) | Oral ivermectin in combination with doxycycline (12 mg ivermectin single dose and 200 mg doxycycline on day 1, followed by 100 mg every 12 h for the next 4 days) | Placebo (n  = 24) | Not specified | 14 | Ratio of VC |
| Aman J et al [20] | 2021 | Netherlands | DRPCTs | RT-PCR | Moderate patients | 385 | Males (n =264);  Females (n =121) | ≥ 18 | Imatinib (n = 197) | 800 mg on day 0 followed by 400 mg daily on days 1–9 | Placebo (n  = 188) | Not specified | 28 | ACM |
| Biber A et al [21] | 2021 | Israel | DRPCTs | RT-PCR | Mild to moderate patients | 89 | Males (n =69); Females (n = 20) | ≥ 18 | Ivermectin (n = 47) | Ivermectin 0·2 mg/kg for 3 days | Placebo (n  = 42) | Not specified | 14 | Ratio of VC; ratio of TEAEs |
| Blum Vf et al [22] | 2021 | Brazil | DRPCTs | RT-PCR | Mild patients | 50 | Males (n =15);  Females (n =35) | ≥ 18 | Nitazoxanide (n = 25) | 600 mg BID for seven days | Placebo (n  = 25) | Not specified | 21 | ACM; ratio of TEAEs |
| Cadegiani FA et al (a) [23] | 2021 | Brazil | DRPCTs | RT-PCR | Mild patients | 236 | Males (n =128);  Females (n =108) | ≥ 18 | Proxalutamide (n = 171) | Proxalutamide 200 mg/day for 15 days or until full COVID-19 remission | Placebo (n  = 65) | Not specified | 15 | Ratio of VC |
| Cadegiani FA et al (b) [24] | 2021 | Brazil | DRPCTs | RT-PCR | Mild to moderate patients | 645 | Males (n =184);  Females (n =133) | ≥ 18 | Proxalutamide (n = 317) | 300 mg of proxalutamide per day for 14 days | Placebo (n  = 328) | Not specified | 28 | ACM; ratio of TEAEs |
| Caricchio R et al [25] | 2021 | Europe and USA | DRPCTs | RT-PCR | Non-severe and hospitalized patients | 454 | Males (n =267);  Females (n =187) | ≥ 18 | Canakinumab (n =227) | 450 mg for body weight of 40-<60 kg, 600 mg for 60-80 kg, and 750 mg for >80 kg | Placebo (n= 227) | Not specified | 29 | ACM; ratio of TEAEs |
| Chaccour C et al [26] | 2021 | Spain | RPCTs | RT-PCR | Non-severe patients | 24 | Males (n = 12); Females (n =12) | 18 to 54 | Ivermectin (n = 12) | 400 mcg/kg | Placebo (n  = 12) | Not specified | 28 | Ratio of VC; ratio of TEAEs |
| Chen J et al [27] | 2020 | China | RPCTs | RT-PCR | Non-severe and hospitalized patients | 134 | Males (n =69);  Females (n =65) | 35-62 | Lopinavir/ritonavir (n = 52) | Lopinavir/ritonavir (2 tablets each time, twice a day, and continuous medication for 5 days) | Placebo (n  = 48) | Not specified | 14 | Ratio of VC; ratio of TEAEs |
| Chen J et al [27] | 2020 | China | RPCTs | RT-PCR | Non-severe and hospitalized patients | 134 | Males (n =69);  Females (n =65) | 35-62 | Arbidol (n = 34) | Arbidol (200 mg each time, three times a day, and continuous medication for 5 days) | Placebo (n  = 48) | Not specified | 14 | Ratio of VC; ratio of TEAEs |

| Devpura G et al [28] | 2021 | India | DRPCTs | RT-PCR | Mild patients | 95 | Males (n =77);  Females (n =18) | ≥ 18 | Ayurvedic (n = 45) | 1 g of Giloy Ghanvati, 2 g of Swasari Ras and 0.5 g each of Ashwagandha and Tulsi Ghanvati twice per day for 7 days | Placebo (n  = 50) | Not specified | 7 | Ratio of VC |
| --- | --- | --- | --- | --- | --- | --- | --- | --- | --- | --- | --- | --- | --- | --- |
| Dubee V et al [29] | 2021 | France, Monaco | MDRPCTs | RT-PCR | Mild to moderate patients | 247 | Males (n =121);  Females (n =129) | ≥ 18 | Hydroxychloroquine (n = 124) | 800mg hydroxychloroquine on Day 0 followed by 400 mg per day for 8 days | Placebo (n  = 123) | Not specified | 28 | ACM; ratio of VC; ratio of TEAEs |
| Eom JS et al [30] | 2021 | Korea | DRPCTs | RT-PCR | Mild to moderate patients | 543 | Males (n =264);  Females (n =279) | ≥ 18 | CT-P59 40 mg/kg (n = 105) | A single dose of CT-P59 40 mg/kg | Placebo (n  = 111) | Not specified | 28 | Ratio of VC; ACM; ratio of TEAEs |
| Eom JS et al [30] | 2021 | Korea | DRPCTs | RT-PCR | Mild to moderate patients | 543 | Males (n =264);  Females (n =279) | ≥ 18 | CT-P59 80 mg/kg (n = 111) | A single dose of CT-P59 80 mg/kg | Placebo (n  = 111) | Not specified | 28 | Ratio of VC; ACM; ratio of TEAEs |
| Eom JS et al [30] | 2021 | Korea | DRPCTs | RT-PCR | Mild to moderate patients | 543 | Males (n =264);  Females (n =279) | ≥ 18 | CT-P59 combined (n = 216) | A single dose of CT-P59 40 mg/kg and 80 mg/kg combined | Placebo (n  = 111) | Not specified | 28 | Ratio of VC; ACM; ratio of TEAEs |
| Feld JJ et al [31] | 2021 | Canada | DRPCTs | RT-PCR | Mild to moderate patients | 60 | Males (n =25);  Females (n =35) | ≥ 18 | Peginterferon lambda (n = 30) | 180 µg within 7 days | Placebo (n  = 30) | Not specified | 7 | Ratio of VC; ratio of TEAEs |
| Gonzalez-Ochoa AG et al [32] | 2021 | Mexico | RPCTs | RT-PCR | Non-severe patients | 243 | Males (n =115); Females (n = 128) | > 40 | Sulodexide (n = 124) | 500 LRU (lipase releasing units) twice daily for 3 weeks | Placebo (n  = 119) | Not specified | 21 | ACM; ratio of TEAEs |
| Gottlieb RL et al [33] | 2021 | USA | MDRPCTs | RT-PCR | Mild to moderate patients | 577 | Males (n =262);  Females (n =315) | ≥ 18 | Low dosage bamlanivimab (n = 101) | 700 mg of bamlanivimab | Placebo (n  = 156) | Not specified | 29 | Ratio of VC; ratio of TEAEs |
| Gottlieb RL et al [33] | 2021 | USA | MDRPCTs | RT-PCR | Mild to moderate patients | 577 | Males (n =262);  Females (n =315) | ≥ 18 | Moderate dosage bamlanivimab (n = 107) | 2800 mg of bamlanivimab | Placebo (n  = 156) | Not specified | 29 | Ratio of VC; ratio of TEAEs |
| Gottlieb RL et al [33] | 2021 | USA | MDRPCTs | RT-PCR | Mild to moderate patients | 577 | Males (n =262);  Females (n =315) | ≥ 18 | High dosage bamlanivimab (n = 101) | 7000 mg of bamlanivimab | Placebo (n  = 156) | Not specified | 29 | Ratio of VC; ratio of TEAEs |
| Gottlieb RL et al [33] | 2021 | USA | MDRPCTs | RT-PCR | Mild to moderate patients | 577 | Males (n =262);  Females (n =315) | ≥ 18 | Moderate dosage bamlanivimab/etesevimab (n = 112) | A combination treatment with 2800 mg of bamlanivimab and 2800 mg of etesevimab | Placebo (n  = 156) | Not specified | 29 | Ratio of VC; ratio of TEAEs |
| Gunst GD et al [34] | 2021 | Sweden and Denmark | MDRPCTs | RT-PCR | Non-severe and hospitalized patients | 205 | Males (n =123); Females (n = 82) | ≥ 18 | Camostat-mesilate (n  =137) | Receive the TMPRSS2 inhibitor camostat-mesilate 200 mg three times daily | Placebo (n  = 68) | Not specified | 30 | ACM; ratio of TEAEs |
| Gupta A et al [35] | 2021 | United States, Canada, Brazil, and Spain | MDRPCTs | RT-PCR | Mild to moderate patients | 1451 | Males (n =266); Females (n = 317) | ≥ 18 | Sotrovimab (n = 721) | A single 500-mg, 1-hour infusion of sotrovimab on day 1 (neutralizing antibody) | Placebo (n  = 730) | A single 500- mg, 1-hour infusion of saline placebo on day 1 | 56 | ACM; ratio of TEAEs |

| Gutierrez-Castrellon P et al [36] | 2021 | Mexico | QRPCTs | RT-PCR | Mild patients | 300 | Males (n =139);  Females (n =161) | 18 to 60 | Novel probiotic formulation (n = 150) | The active product consisted of a blend of four strains of freeze-dried lactic acid bacteria: Lactiplantibacillus plantarum KABP033 (CECT30292), L.  plantarum KABP022 (CECT7484),  L. plantarum KABP023 (CECT7485)) and Pediococcus acidilactici KABP021 (CECT7483) | Placebo (n  = 150) | Placebo product consisted of HPMC  capsules containing the maltodextrin carrier only | 30 | ACM; ratio of TEAEs |
| --- | --- | --- | --- | --- | --- | --- | --- | --- | --- | --- | --- | --- | --- | --- |
| Humeniuk R et al [37] | 2020 | American | DRPCTs | RT-PCR | Non-severe patients | 96 | Males (n =56);  Females (n =40) | 18 to 55 | Remdesivir (n = 8) | Receive Remdesivir solution formulation at doses ranging from 3 mg to 225 mg | Placebo (n  = 18) | Not specified | 43 | Ratio of TEAEs |
| Jagannathan P et al [38] | 2021 | USA | RPCTs | RT-PCR | Non-severe patients | 120 | Males (n =50);  Females (n =70) | 18 to 55 | Peginterferon lambda (n = 60) | Those assigned to Lambda received a single 180 mcg subcutaneous injection of study drug (0.45 ml volume) | Placebo (n  = 60) | Placebo received a 0.45 ml subcutaneous injection of saline | 28 | Ratio of TEAEs |
| Jeronimo CMP et al [39] | 2021 | Brazil | DRPCTs | RT-PCR | Non-severe and hospitalized patients | 393 | Males (n =50);  Females (n =70) | ≥ 18 | Methylprednisolone (n = 194) | 0.5 mg/kg twice daily for 5 days | Placebo (n  = 199) | Not specified | 28 | ACM; ratio of VC |
| Lenze EJ et al [40] | 2020 | USA | DRPCTs | RT-PCR | Mild to moderate patients | 152 | Males (n =43);  Females (n =109) | ≥ 18 | Fluvoxamine (n = 80) | Fluvoxamine 50mg, then for 2 days at a dose of 100 mg twice daily as tolerated, and then increasing to a dose of 100 mg 3 times daily as tolerated through day 15 | Placebo (n  = 72) | Not specified | 30 | Ratio of TEAEs |
| López-Medina E et al [41] | 2021 | Colombia | DRPCTs | RT-PCR | Mild patients | 398 | Males (n =167);  Females (n =231) | ≥ 18 | Ivermectin (n = 200) | Receive ivermectin,300 μg/kg of body weight per day for 5 days | Placebo (n  = 198) | Receive placebo, 300 μg/kg of body weight per day for 5 days | 21 | ACM; ratio of TEAEs |
| Marconi VC et al [42] | 2021 | Asia, Europe, North America, and South America | MDRPCTs | RT-PCR | Mild to moderate patients | 1525 | Males (n = 963); Females (n =562) | ≥ 18 | Baricitinib (n = 764) | Baricitinib 4-mg or 2-mg daily | Placebo (n  = 761) | Not specified | 28 | ACM; ratio of TEAEs |
| McCoy J et al [43] | 2021 | Brazil | DRPCTs | RT-PCR | Mild patients | 268 | Males (n =268);  Females (n =0) | ≥ 18 | Proxalutamide (n = 134) | Proxalutamide 200mg/day or placebo for up to 7 days | Placebo (n  = 134) | Not specified | 30 | ACM; ratio of TEAEs |

| Mohan A et al [44] | 2020 | India | DRPCTs | RT-PCR | Non-severe and hospitalized patients | 125 | Males (n =80);  Females (n =45) | ≥ 18 | Ivermectin (n = 40) | A single oral administration of Ivermectin elixir at 24 mg | Placebo (n  = 45) | 1:1 ratio | 28 | Ratio of VC; ratio of TEAEs |
| --- | --- | --- | --- | --- | --- | --- | --- | --- | --- | --- | --- | --- | --- | --- |
| Mohan A et al [44] | 2020 | India | DRPCTs | RT-PCR | Non-severe and hospitalized patients | 125 | Males (n =80);  Females (n =45) | ≥ 18 | Ivermectin (n = 40) | A single oral administration of Ivermectin elixir at 12 mg | Placebo (n  = 45) | 1:1 ratio | 28 | Ratio of VC; ratio of TEAEs |
| Monk PD et al [45] | 2020 | UK | DRPCTs | RT-PCR | Non-severe and hospitalized patients | 101 | Males (n =58);  Females (n =40) | ≥ 18 | Interferon beta (n = 50) | Receive inhaled nebulised interferon beta-1a (SNG001) (6 MIU) | Placebo (n  =51) | Receive placebo (6 MIU) | 60 | ACM; ratio of TEAEs |
| Omrani AS et al [46] | 2020 | United States | RPCTs | RT-PCR | Non-severe and hospitalized patients | 452 | Males (n =449);  Females (n =7) | ≥ 18 | Hydroxychloroquine (n = 152) | Oral hydroxychloroquine (600 mg daily for one week) | Placebo (n  = 152) | Not specified | 14 | Ratio of VC |
| Omrani AS et al [46] | 2020 | United States | RPCTs | RT-PCR | Mild and hospitalized patients | 452 | Males (n =449);  Females (n =7) | ≥ 18 | Hydroxychloroquine/azithr omycin (n = 152) | Oral hydroxychloroquine plus oral azithromycin (500 mg day one,250 mg daily on days two through five) | Placebo (n  = 152) | Not specified | 14 | Ratio of VC |
| Patel O et al [47] | 2021 | Australia | DRPCTs | RT-PCR | Non-severe and hospitalized patients | 33 | Males (n = 21); Females (n =12) | ≥ 18 | High-dose intravenous zinc (HDIVZn) (n =15) | A dose of 0.5 mg/kg/day (elemental zinc concentration, 0.24 mg/kg/day) for a maximum of 7 days, or until hospital discharge or death | Placebo (n  = 18) | Not specified | 28 | ACM |
| Puskarich MA et al [48] | 2021 | USA | MDRPCTs | RT-PCR | Mild patients | 117 | Males (n =59);  Females (n =58) | ≥ 18 | Losartan (n = 58) | 25 mg orally twice daily | Placebo (n  = 59) | Not specified | 28 | ACM |
| Rastogi A et al [49] | 2020 | India | RPCTs | RT-PCR | Mild patients | 40 | Males (n =20); Females (n = 20) | ≥ 18 | High-dose vitamin D (n = 16) | Daily 60 000 IU of vitamin D (oral nano-liquid droplets) for 7 days with therapeutic target 25(OH) D  >50 ng/ml | Placebo (n  = 24) | 5 ml distilled  water for 7 days | 21 | Ratio of VC |
| Ravikirti et al [50] | 2021 | India | RPCTs | RT-PCR | Mild to moderate patients | 115 | Males (n =81); Females (n = 31) | ≥ 18 | Ivermectin (n = 55) | Ivermectin 12 mg on day 1 and day admission | Placebo (n  = 57) | Given identical looking placebo tablets | 10 | Ratio of VC; ACM |
| Reis G et al [51] | 2021 | Brazil | RPCTs | RT-PCR | Mild patients | 735 | Males (n = 308); Females (n = 377) | ≥ 18 | Hydroxychloroquine (n = 214) | Hydroxychloroquine (800 mg loading dose, then 400 mg daily for 9 days) | Placebo (n  = 277) | Not specified | 90 | Ratio of TEAEs |
| Reis G et al [51] | 2021 | Brazil | RPCTs | RT-PCR | Mild patients | 735 | Males (n = 308); Females (n = 377) | ≥ 18 | Lopinavir/ritonavir (n = 244) | Lopinavir/ritonavir (loading dose of 800 mg and 200 mg, respectively, every 12 hours followed by 400 mg and 100 mg, respectively, every 12 hours for the next 9 days) | Placebo (n  = 277) | Not specified | 90 | Ratio of TEAEs |
| Rocco PRM et al [52] | 2020 | Brazil | DRPCTs | RT-PCR | Mild patients | 392 | Males (n =184); Females (n = 208) | ≥ 18 | Nitazoxanide (n = 194) | Nitazoxanide (500 mg oral solution, three times daily for 5 days) | Placebo (n  = 198) | Not specified | 14 | Ratio of VC; ratio of TEAEs |

| Salama C et al [53] | 2021 | USA | DRPCTs | RT-PCR | Mild to moderate patients | 377 | Males (n =223); Females (n = 154) | ≥ 18 | Tocilizumab (n = 249) | Tocilizumab 8 mg per kilogram of body weight intravenously | Placebo (n  = 128) | Not specified | 60 | ACM; ratio of TEAEs |
| --- | --- | --- | --- | --- | --- | --- | --- | --- | --- | --- | --- | --- | --- | --- |
| Silva M et al [54] | 2021 | Argentina | DRPCTs | RT-PCR | Mild patients | 40 | Males (n =26);  Females (n =10) | ≥ 18 | Nitazoxanide (n = 27) | 3 g/day then 2 g/day | Placebo (n  = 13) | Not specified | 7 | ACM; ratio of VC; ratio of TEAEs |
| Sivapalan P et al [55] | 2021 | Denmark | DRPCTs | RT-PCR | Non-severe and hospitalized patients | 117 | Males (n =65); Females (n = 51) | ≥ 18 | Hydroxychloroquine/azithr omycin (n = 61) | Azithromycin (500 mg for 3 days  and 250 mg for 12 days)  +hydroxychloroquine (200 mg twice daily) | Placebo (n  = 56) | Not specified | 14 | ACM |
| Skipper CP et al [56] | 2020 | United States and Canada | MDRPCTs | RT-PCR | Non-severe patients | 423 | Males (n = 185); Females (n =238) | ≥ 18 | Hydroxychloroquine  +standard of care (n =212) | Hydroxychloroquine 800 mg once, then 600 mg 6-8 hours later, then 600 mg once daily for 4 days | Placebo (n  = 211) | Not specified | 60 | ACM; ratio of TEAEs |
| Stone JH et al [57] | 2020 | USA | DRPCTs | RT-PCR or  serum IgM | Mild to moderate patients | 243 | Males (n = 141); Females (n =102) | ≥ 18 | Tocilizumab (n = 161) | Tocilizumab 8 mg per kilogram of body weight | Placebo (n  = 82) | Not specified | 28 | ACM; ratio of TEAEs |
| Tardif JC et al [58] | 2021 | Brazil, Canada, Greece, South Africa, Spain, and the USA | MDRPCTs | RT-PCR | Mild patients | 4488 | Males (n =148);  Females (n =87) | ≥ 40 | Colchicine (n = 2235) | Colchicine (0·5 mg twice per day for the first 3 days and then once per day for 27 days thereafter) | Placebo (n  = 2253) | Not specified | 30 | ACM; ratio of TEAEs |
| Tornling G et al [59] | 2021 | India | DRPCTs | RT-PCR | Non-severe and hospitalized patients | 106 | Males (n = 80); Females (n =26) | 19 to 69 | Angiotensin II type 2 receptor agonist C21 (n = 51) | Oral C21 (100 mg twice daily) | Placebo (n  = 55) | Not specified | 14 | ACM |
| Ulrich RJ et al [60] | 2020 | United States | MDRPCTs | RT-PCR | Non-severe and hospitalized patients | 128 | Males (n = 76); Females (n =52) | ≥ 18 | Hydroxychloroquine (n = 67) | 400 mg (2 tablets) by mouth 2 times  per day (day 1) and 200 mg (1 tablet) by mouth 2 times per day (days 2–5) | Placebo (n  = 61) | The dose likes the intervention group | 30 | Ratio of VC; ACM; ratio of TEAEs |
| Vallejos J et al [61] | 2021 | Argentina | DRPCTs | RT-PCR | Mild patients | 501 | Males (n =237);  Females (n =264) | ≥ 18 | Ivermectin (n = 250) | 24 mg daily (less than 80 kg); 36  mg daily (80 to 110 kg); 48 mg daily (more than 110kg) | Placebo (n  = 251) | Not specified | 35 | ACM; ratio of VC; ratio of TEAEs |
| Wang Y et al [62] | 2020 | China | DRPCTs | RT-PCR | Non-severe and hospitalized patients | 237 | Males (n =140);  Females (n =96) | ≥ 18 | Remdesivir (n = 158) | Intravenous remdesivir (200 mg on day 1 followed by 100 mg on days 2–10 in single daily infusions) | Placebo (n  = 79) | The same volume of placebo infusions for 10 days | 28 | Ratio of VC; ACM; ratio of TEAEs |
| Weinreich DM et al [63] | 2021 | USA | DRPCTs | RT-PCR | Mild to moderate patients | 269 | Males (n =134);  Females (n =141) | ≥ 18 | REGN-COV2  (neutralizing monoclonal antibodies) (n = 176) | REGN-COV2 at a dose of 2.4 g (low dose), or REGN-COV2 at a dose of 8.0 g (high dose) | Placebo (n  = 93) | Not specified | 29 | Ratio of VC; ratio of TEAEs |
| CaoY et al [64] | 2020 | China | RPCTs | RT-PCR | Severe and hospitalized patients | 41 | Males (n =24);  Females (n =17) | ≥ 18 | Ruxolitinib (n = 20) | 5 mg twice a day | Placebo (n  = 21) | Not specified | 28 | ACM; ratio of TEAEs |

| Cremer PC et al [65] | 2021 | USA | MDRPCTs | RT-PCR | Severe and hospitalized patients | 40 | Males (n =26); Females (n = 14) | ≥ 18 | Mavrilimumab (n = 21) | Receive mavrilimumab 6 mg/kg as a single intravenous infusion | Placebo (n  = 198) | Not specified | 28 | ACM |
| --- | --- | --- | --- | --- | --- | --- | --- | --- | --- | --- | --- | --- | --- | --- |
| de Alencar JCG et al [66] | 2021 | Brazil | DRPCTs | RT-PCR | Severe and hospitalized patients | 135 | Males (n = 80); Females (n =55) | ≥ 18 | N-acetylcysteine (NAC, n  = 67) | N-acetylcysteine (NAC,28 mg/mL and 14 mg/mL) | Placebo (n  = 68) | Dextrose 5% in  water (1000 mL in total) intravenously | 9 | ACM |
| Dequin PF et al [67] | 2020 | France | DRPCTs | RT-PCR | Severe and hospitalized patients | 149 | Males (n =104);  Females (n =45) | ≥ 18 | Hydrocortisone (n = 76) | 200 mg/d until day 7, 100 mg/d for 4 days and 50 mg/d for 3 days, for a total of 14 days | Placebo (n  = 73) | Not specified | 14 | ACM |
| Gharebaghi N et al [68] | 2020 | Iran | DRPCTs | RT-PCR | Severe and hospitalized patients | 59 | Males (n =41);  Females (n =18) | ≥ 18 | Immunoglobulin gamma (n  = 30) | IVIg (human) flebogamma 5% DIF GRIFOLS daily for three consecutive days | Placebo (n  = 29) | Not specified | 28 | ACM |
| Lescure FX et al [69] | 2021 | Argentina, Brazil, Canada, Chile, France, Germany, Israel, Italy, Japan, Russia, and Spain | MDRPCTs | RT-PCR | Severe and hospitalized patients | 416 | Males (n = 261); Females (n =155) | ≥ 18 | Sarilumab 200 mg (n = 159) | Intravenous sarilumab 200 mg | Placebo (n  = 84) | Not specified | 60 | ACM; ratio of TEAEs |
| Lescure FX et al [69] | 2021 | Argentina, Brazil, Canada, Chile, France, Germany, Israel, Italy, Japan, Russia, and Spain | MDRPCTs | RT-PCR | Severe and hospitalized patients | 416 | Males (n = 261); Females (n =155) | ≥ 18 | Sarilumab 400 mg (n = 173) | Intravenous sarilumab 400 mg | Placebo (n  = 84) | Not specified | 60 | ACM; ratio of TEAEs |
| Libster R et al [70] | 2021 | Argentina | DRPCTs | RT-PCR | Severe and hospitalized patients | 160 | Males (n = 60); Females (n =100) | ≥ 65 | Convalescent Plasma (n = 80) | Convalescent plasma 250ml with an IgG titer greater than 1:1000 | Placebo (n  = 80) | Not specified | 25 | ACM |
| Munch MW et al [71] | 2021 | Denmark, Sweden, Switzerland and India | MDRPCTs | RT-PCR | Severe and hospitalized patients | 30 | Males (n =24); Females (n = 6) | ≥ 18 | Hydrocortisone (n = 16) | Hydrocortisone 200mg per day for 7days or until discharge | Placebo (n  = 14) | Not specified | 90 | ACM |

| Rosas IO et al [72] | 2021 | Europe and North America | MDRPCTs | RT-PCR | Severe and hospitalized patients | 438 | Males (n =306);  Females (n =132) | ≥ 18 | Tocilizumab (n = 294) | 8 mg per kilogram of body weight, with a maximum dose of 800 mg | Placebo (n  = 144) | Not specified | 28 | ACM; ratio of TEAEs |
| --- | --- | --- | --- | --- | --- | --- | --- | --- | --- | --- | --- | --- | --- | --- |
| Sehgal IS et al [73] | 2021 | India | DRPCTs | RT-PCR | Severe and hospitalized patients | 42 | Males (n =29);  Females (n =13) | ≥ 18 | Mycobacterium-w (n = 20) | Each dose of 0.1 mL Mw contains 0.5×109 heat killed Mycobacterium w, 0.9% sodium chloride, and 0.01% thimerosal (as preservative) | Placebo (n  = 22) | 0.9% sodium chloride, 0.01%  thiomersal | 28 | ACM; ratio of TEAEs |
| Shi L et al [74] | 2021 | China | DRPCTs | RT-PCR | Severe and hospitalized patients | 100 | Males (n =56);  Females (n =44) | ≥ 18 | Human umbilical cord- derived mesenchymal stem cells (UC-MSCs) (n = 65) | UC-MSCs (4 × 107 cells per infusion) | Placebo (n  = 35) | Not specified | 28 | Ratio of TEAEs |
| Simonovich VA et al [75] | 2020 | Argentina | RPCTs | RT-PCR | Severe and hospitalized patients | 333 | Males (n =225);  Females (n =108) | ≥ 18 | Convalescent Plasma (n = 228) | Received convalescent plasma | Placebo (n  = 105) | Not specified | 30 | ACM; ratio of TEAEs |
| Sivapalasingam S et al [76] | 2021 | USA | DRPCTs | RT-PCR | Severe and hospitalized patients | 126 | Males (n =531);  Females (n =219) | ≥ 18 | Sarilumab 200 mg group (n = 50) | Intravenous sarilumab 200 mg | Placebo (n  = 25) | Not specified | 22 | ACM; ratio of TEAEs |
| Sivapalasingam S et al [76] | 2021 | USA | DRPCTs | RT-PCR | Severe and hospitalized patients | 126 | Males (n =531);  Females (n =219) | ≥ 18 | Sarilumab 400 mg group (n = 51) | Intravenous sarilumab 400 mg | Placebo (n  = 25) | Not specified | 22 | ACM; ratio of TEAEs |
| Temesgen Z et al [77] | 2021 | USA and Brazil | DRPCTs | RT-PCR | Severe and hospitalized patients | 479 | Males (n =310);  Females (n =169) | ≥ 18 | Lenzilumab (n = 236) | Three doses of lenzilumab (600 mg, each) or placebo were administered 8 hours apart via a 1-hour IV infusion | Placebo (n  = 243) | Not specified | 28 | ACM; ratio of TEAEs |
| Zhong M et al [78] | 2020 | China | RPCTs | RT-PCR | Severe and hospitalized patients | 17 | Males (n =13 ); Females (n = 4) | 51 to 91 | α-Lipoic acid (n = 8) | 1200 mg/d, intravenous infusion | Placebo (n  = 9) | Equal volume saline infusion (placebo) for 7 days | 30 | ACM |
| *If the total number of genders is not equal to the number of participants, it is due to loss to follow-up from participants. RPCTs, randomised placebo-controlled trials; COVID-19, coronavirus disease 2019; DRPCTs, double-blind, randomised placebo-controlled trials; QRPCTs, quadruple-blinded, randomised placebo-controlled trials; MDRPCTs, multicenter, double-blind, randomised placebo-controlled trials; RT-PCR, reverse transcription-polymerase chain reaction; VC, virological cure; ACM, all-cause mortality; TEAEs, treatment-emergent adverse events. | | | | | | | | | | | | | | |
