# Supplementary material for: Efficacy and safety of current medications for treating severe and non-severe COVID-19 patients: an updated network meta-analysis of randomized placebo-controlled trials
Source: Aging (Albany NY). 2021 Sep 16;13(18):21866–902. doi: 10.18632/aging.203522 (PMC8507270; doi:10.18632/aging.203522)
Supplement: Supplementary Table 2 [file aging-13-203522-s003.pdf]

Supplementary Table 2. Rating of individual items of the Risk of Bias tool for each study.

Note: CSR: Clinical Study Report

ACTIV-3/TICO LY-CoV555 Study Group [18]

ITEM	RATING	SUPPORT
**Random sequence generation**	LOW	Received medications and placebo in a random order, double-blind .
**Allocation concealment**	LOW	Information from full CSR, Method of Assigning Subjects to Treatment Groups.
**Blinding participants/personnel**	LOW	"Investigator and the patient were blinded to treatment. To maintain blinding, all investigational products were supplied." Information from full CSR, Blinding.
**Blinding assessor**	LOW	"Investigator and the patient were blinded to treatment. To maintain blinding, all investigational products were supplied." Information from full CSR, Blinding.
**Incomplete data outcome**	LOW	Outcomes relevant for the present meta-analysis mentioned in the full CSR were reported in the Journal article.
**Selective reporting**	UNCLEAR	No details on selective reporting.
**Other bias**	LOW	Outcomes relevant for the present meta-analysis mentioned in the full CSR were reported in the Journal article.
**Notes**	First author contacted but not able to provide additional information.	

Ahmed S et al [19]

ITEM	RATING	SUPPORT
**Random sequence generation**	LOW	Received medications and placebo in a random order, double-blind .
**Allocation concealment**	LOW	Information from full CSR, Method of Assigning Subjects to Treatment Groups.
**Blinding participants/personnel**	LOW	“Investigator and the patient were blinded to treatment. To maintain blinding, all investigational products were supplied.“ Information from full CSR, Blinding.
**Blinding assessor**	LOW	“Investigator and the patient were blinded to treatment. To maintain blinding, all investigational products were supplied.“ Information from full CSR, Blinding.
**Incomplete data outcome**	LOW	Outcomes relevant for the present meta-analysis mentioned in the full CSR were reported in the Journal article.
**Selective reporting**	UNCLEAR	No details on selective reporting.
**Other bias**	UNCLEAR	No details on other bias.
**Notes**	Authors contacted but no reply.	

Aman J et al [20]

ITEM	RATING	SUPPORT
**Random sequence generation**	LOW	Received medications and placebo in a random order, double-blind .
**Allocation concealment**	LOW	Information from full CSR, Method of Assigning Subjects to Treatment Groups.
**Blinding participants/personnel**	LOW	“Investigator and the patient were blinded to treatment. To maintain blinding, all investigational products were supplied.“ Information from full CSR, Blinding.

**Blinding assessor**	LOW	"Investigator and the patient were blinded to treatment. To maintain blinding, all investigational products were supplied." Information from full CSR, Blinding.
**Incomplete data outcome**	LOW	Outcomes relevant for the present meta-analysis mentioned in the full CSR were reported in the Journal article.
**Selective reporting**	UNCLEAR	No details on selective reporting.
**Other bias**	UNCLEAR	No details on other bias.
**Notes**	First author contacted but not able to provide additional information.	

Biber A et al [21]

**ITEM**	**RATING**	**SUPPORT**
**Random sequence generation**	LOW	Received medications and placebo in a random order, double-blind .
**Allocation concealment**	LOW	Information from full CSR, Method of Assigning Subjects to Treatment Groups.
**Blinding participants/personnel**	LOW	"Investigator and the patient were blinded to treatment. To maintain blinding, all investigational products were supplied." Information from full CSR, Blinding.
**Blinding assessor**	LOW	"Investigator and the patient were blinded to treatment. To maintain blinding, all investigational products were supplied." Information from full CSR, Blinding.
**Incomplete data outcome**	LOW	Outcomes relevant for the present meta-analysis mentioned in the full CSR were reported in the Journal article.
**Selective reporting**	UNCLEAR	No details on selective reporting.
**Other bias**	UNCLEAR	No details on other bias.
**Notes**	Authors contacted but no reply.	

Blum Vf et al [22]

**ITEM**	**RATING**	**SUPPORT**
**Random sequence generation**	LOW	Received medications and placebo in a random order, double-blind .
**Allocation concealment**	LOW	Information from full CSR, Method of Assigning Subjects to Treatment Groups.
**Blinding participants/personnel**	LOW	"Investigator and the patient were blinded to treatment. To maintain blinding, all investigational products were supplied." Information from full CSR, Blinding.
**Blinding assessor**	LOW	"Investigator and the patient were blinded to treatment. To maintain blinding, all investigational products were supplied." Information from full CSR, Blinding.
**Incomplete data outcome**	UNCLEAR	No details on incomplete data outcome.
**Selective reporting**	LOW	Outcomes listed in the CSR synopsis reported in the journal article.
**Other bias**	UNCLEAR	No details on other bias.
**Notes**	Not possible to contact authors.	

Cadegiani FA et al (a) [23]

**ITEM**	**RATING**	**SUPPORT**
**Random sequence generation**	LOW	Received medications and placebo in a random order, double-blind .

**Allocation concealment**	LOW	Information from full CSR, Method of Assigning Subjects to Treatment Groups.
**Blinding participants/personnel**	LOW	“Investigator and the patient were blinded to treatment. To maintain blinding, all investigational products were supplied.“ Information from full CSR, Blinding.
**Blinding assessor**	LOW	“Investigator and the patient were blinded to treatment. To maintain blinding, all investigational products were supplied.“ Information from full CSR, Blinding.
**Incomplete data outcome**	LOW	Outcomes relevant for the present meta-analysis mentioned in the full CSR were reported in the Journal article.
**Selective reporting**	LOW	First author confirmed that all measures listed in the protocol are reported in the journal article.
**Other bias**	UNCLEAR	No details on other bias.
**Notes**	First author provided additional information.	

Cadegiani FA et al (b) [24]

**ITEM**	**RATING**	**SUPPORT**
**Random sequence generation**	LOW	Received medications and placebo in a random order, double-blind .
**Allocation concealment**	LOW	Information from full CSR, Method of Assigning Subjects to Treatment Groups.
**Blinding participants/personnel**	LOW	“Investigator and the patient were blinded to treatment. To maintain blinding, all investigational products were supplied.“ Information from full CSR, Blinding.
**Blinding assessor**	LOW	“Investigator and the patient were blinded to treatment. To maintain blinding, all investigational products were supplied.“ Information from full CSR, Blinding.
**Incomplete data outcome**	LOW	Outcomes relevant for the present meta-analysis mentioned in the full CSR were reported in the Journal article.
**Selective reporting**	UNCLEAR	No details on selective reporting.
**Other bias**	UNCLEAR	No details on other bias.
**Notes**	First author informed that additional data are not available anymore.	

Caricchio R et al [25]

**ITEM**	**RATING**	**SUPPORT**
**Random sequence generation**	LOW	Received medications and placebo in a random order, double-blind .
**Allocation concealment**	LOW	Information from full CSR, Method of Assigning Subjects to Treatment Groups.
**Blinding participants/personnel**	LOW	“Investigator and the patient were blinded to treatment. To maintain blinding, all investigational products were supplied.“ Information from full CSR, Blinding.
**Blinding assessor**	LOW	“Investigator and the patient were blinded to treatment. To maintain blinding, all investigational products were supplied.“ Information from full CSR, Blinding.
**Incomplete data outcome**	LOW	Outcomes relevant for the present meta-analysis mentioned in the full CSR were reported in the Journal article.
**Selective reporting**	UNCLEAR	No details on selective reporting.
**Other bias**	UNCLEAR	No details on other bias.
**Notes**	First author contacted but not able to provide additional information.	

Chaccour C et al [26]

ITEM	RATING	SUPPORT
**Random sequence generation**	LOW	Received medications and placebo in a random order, double-blind .
**Allocation concealment**	LOW	Information from the Journal article, Method of Assigning Subjects to Treatment Groups.
**Blinding participants/personnel**	UNCLEAR	No protocol/CSR available.
**Blinding assessor**	UNCLEAR	No protocol/CSR available.
**Incomplete data outcome**	LOW	Outcomes relevant for the present meta-analysis mentioned were reported in the Journal article.
**Selective reporting**	LOW	Information provided by the manufacturer: some outcomes were not reported in the journal article (however, available from Clinicaltrials.gov.)
**Other bias**	UNCLEAR	No protocol/CSR available.
**Notes**	Manufacturer provided additional information.	

Chen J et al [27]

ITEM	RATING	SUPPORT
**Random sequence generation**	LOW	Received medications and placebo in a random order, double-blind .
**Allocation concealment**	LOW	Information from the Journal article, Method of Assigning Subjects to Treatment Groups.
**Blinding participants/personnel**	UNCLEAR	No protocol/CSR available.
**Blinding assessor**	LOW	“Investigator and the patient were blinded to treatment. To maintain blinding, all investigational products were supplied.“ Information from the Journal article, Blinding.
**Incomplete data outcome**	LOW	Outcomes relevant for the present meta-analysis mentioned were reported in the Journal article.
**Selective reporting**	UNCLEAR	No protocol/CSR available.
**Other bias**	UNCLEAR	No protocol/CSR available.
**Notes**	First author contacted but not able to provide additional information.	

Devpura G et al [28]

ITEM	RATING	SUPPORT
**Random sequence generation**	LOW	Received medications and placebo in a random order, double-blind .
**Allocation concealment**	LOW	Information from full CSR, Method of Assigning Subjects to Treatment Groups.
**Blinding participants/personnel**	LOW	“Investigator and the patient were blinded to treatment. To maintain blinding, all investigational products were supplied.“ Information from full CSR, Blinding.
**Blinding assessor**	LOW	“Investigator and the patient were blinded to treatment. To maintain blinding, all investigational products were supplied.“ Information from full CSR, Blinding.
**Incomplete data outcome**	UNCLEAR	No details on incomplete data outcome.
**Selective reporting**	LOW	Information provided by the manufacturer: some outcomes were not reported in the journal article (however, available from Clinicaltrials.gov.)
**Other bias**	UNCLEAR	No details on other bias.
**Notes**	Authors provided additional information.	

Dubee V et al [29]

ITEM	RATING	SUPPORT
**Random sequence generation**	LOW	Received medications and placebo in a random order, double-blind .
**Allocation concealment**	LOW	Information from full CSR, Method of Assigning Subjects to Treatment Groups.
**Blinding participants/personnel**	LOW	“Investigator and the patient were blinded to treatment. To maintain blinding, all investigational products were supplied.“ Information from full CSR, Blinding.
**Blinding assessor**	LOW	“Investigator and the patient were blinded to treatment. To maintain blinding, all investigational products were supplied.“ Information from full CSR, Blinding.
**Incomplete data outcome**	LOW	Outcomes relevant for the present meta-analysis mentioned in the full CSR were reported in the Journal article.
**Selective reporting**	LOW	Information provided by the manufacturer: some outcomes were not reported in the journal article (however, available from Clinicaltrials.gov.)
**Other bias**	UNCLEAR	No details on other bias.
**Notes**	Authors provided additional information.	

Eom JS et al [30]

ITEM	RATING	SUPPORT
**Random sequence generation**	LOW	Received medications and placebo in a random order, double-blind .
**Allocation concealment**	LOW	Information from full CSR, Method of Assigning Subjects to Treatment Groups.
**Blinding participants/personnel**	LOW	“Investigator and the patient were blinded to treatment. To maintain blinding, all investigational products were supplied.“ Information from full CSR, Blinding.
**Blinding assessor**	LOW	“Investigator and the patient were blinded to treatment. To maintain blinding, all investigational products were supplied.“ Information from full CSR, Blinding.
**Incomplete data outcome**	LOW	Outcomes relevant for the present meta-analysis mentioned in the full CSR were reported in the Journal article.
**Selective reporting**	UNCLEAR	No details on selective reporting.
**Other bias**	UNCLEAR	No details on other bias.
**Notes**	First author contacted but not able to provide additional information.	

Feld JJ et al [31]

ITEM	RATING	SUPPORT
**Random sequence generation**	LOW	Received medications and placebo in a random order, double-blind .
**Allocation concealment**	LOW	Information from full CSR, Method of Assigning Subjects to Treatment Groups.
**Blinding participants/personnel**	LOW	“Investigator and the patient were blinded to treatment. To maintain blinding, all investigational products were supplied.“ Information from full CSR, Blinding.
**Blinding assessor**	LOW	“Investigator and the patient were blinded to treatment. To maintain blinding, all investigational products were supplied.“ Information from full CSR, Blinding.

**Incomplete data outcome**	LOW	Outcomes relevant for the present meta-analysis mentioned in the full CSR were reported in the Journal article.
**Selective reporting**	LOW	Primary and secondary outcomes listed in full CSR all reported in the journal article.
**Other bias**	UNCLEAR	No details on other bias.
**Notes**	Authors provided additional information.	

Gonzalez-Ochoa AG et al [32]

**ITEM**	**RATING**	**SUPPORT**
**Random sequence generation**	LOW	Received medications and placebo in a random order, double-blind .
**Allocation concealment**	LOW	Information fromhe Journal article, Method of Assigning Subjects to Treatment Groups.
**Blinding participants/personnel**	HIGH	No information on how blinding was preserved.
**Blinding assessor**	HIGH	No information on how blinding was preserved.
**Incomplete data outcome**	LOW	Outcomes relevant for the present meta-analysis mentioned were reported in the Journal article.
**Selective reporting**	UNCLEAR	No protocol/CSR available.
**Other bias**	UNCLEAR	No protocol/CSR available.
**Notes**	First author contacted but not able to provide additional information.	

Gottlieb RL et al [33]

**ITEM**	**RATING**	**SUPPORT**
**Random sequence generation**	LOW	Received medications and placebo in a random order, double-blind .
**Allocation concealment**	LOW	Information from full CSR, Method of Assigning Subjects to Treatment Groups.
**Blinding participants/personnel**	LOW	“Investigator and the patient were blinded to treatment. To maintain blinding, all investigational products were supplied.“ Information from full CSR, Blinding.
**Blinding assessor**	LOW	“Investigator and the patient were blinded to treatment. To maintain blinding, all investigational products were supplied.“ Information from full CSR, Blinding.
**Incomplete data outcome**	LOW	Outcomes relevant for the present meta-analysis mentioned in the full CSR were reported in the Journal article.
**Selective reporting**	LOW	Manufacturer confirmed that “All outcomes were reported in published papers”.
**Other bias**	UNCLEAR	No details on other bias.
**Notes**	Authors provided additional information.	

Gunst GD et al [34]

**ITEM**	**RATING**	**SUPPORT**
**Random sequence generation**	LOW	Received medications and placebo in a random order, double-blind .
**Allocation concealment**	LOW	Information from full CSR, Method of Assigning Subjects to Treatment Groups.
**Blinding participants/personnel**	LOW	“Investigator and the patient were blinded to treatment. To maintain blinding, all investigational products were supplied.“ Information from full CSR, Blinding.

**Blinding assessor**	LOW	"Investigator and the patient were blinded to treatment. To maintain blinding, all investigational products were supplied.“ Information from full CSR, Blinding.
**Incomplete data outcome**	LOW	Outcomes relevant for the present meta-analysis mentioned in the full CSR were reported in the Journal article.
**Selective reporting**	LOW	Manufacturer confirmed that "All outcomes were reported in published papers".
**Other bias**	UNCLEAR	No details on other bias.
**Notes**	Authors provided additional information.	

Gupta A et al [35]

ITEM	RATING	SUPPORT
**Random sequence generation**	LOW	Received medications and placebo in a random order, double-blind .
**Allocation concealment**	LOW	Information from full CSR, Method of Assigning Subjects to Treatment Groups.
**Blinding participants/personnel**	LOW	"Investigator and the patient were blinded to treatment. To maintain blinding, all investigational products were supplied.“ Information from full CSR, Blinding.
**Blinding assessor**	LOW	"Investigator and the patient were blinded to treatment. To maintain blinding, all investigational products were supplied.“ Information from full CSR, Blinding.
**Incomplete data outcome**	LOW	Outcomes relevant for the present meta-analysis mentioned in the full CSR were reported in the Journal article.
**Selective reporting**	LOW	Outcomes relevant for the present meta-analysis mentioned in the CSR were reported in the Journal article.
**Other bias**	UNCLEAR	No details on other bias.
**Notes**	First author provided additional information.	

Gutierrez-Castrellon P et al [36]

ITEM	RATING	SUPPORT
**Random sequence generation**	LOW	Received medications and placebo in a random order, double-blind .
**Allocation concealment**	LOW	Information from full CSR, Method of Assigning Subjects to Treatment Groups.
**Blinding participants/personnel**	LOW	"Investigator and the patient were blinded to treatment. To maintain blinding, all investigational products were supplied.“ Information from full CSR, Blinding.
**Blinding assessor**	LOW	"Investigator and the patient were blinded to treatment. To maintain blinding, all investigational products were supplied.“ Information from full CSR, Blinding.
**Incomplete data outcome**	LOW	Outcomes relevant for the present meta-analysis mentioned in the full CSR were reported in the Journal article.
**Selective reporting**	UNCLEAR	No details on selective reporting.
**Other bias**	UNCLEAR	No details on other bias.
**Notes**	Author contacted but no reply.	

Humeniuk R et al [37]

ITEM	RATING	SUPPORT

**Random sequence generation**	LOW	Received medications and placebo in a random order, double-blind .
**Allocation concealment**	LOW	Information from full CSR, Method of Assigning Subjects to Treatment Groups.
**Blinding participants/personnel**	LOW	“Investigator and the patient were blinded to treatment. To maintain blinding, all investigational products were supplied.“ Information from full CSR, Blinding.
**Blinding assessor**	LOW	“Investigator and the patient were blinded to treatment. To maintain blinding, all investigational products were supplied.“ Information from full CSR, Blinding.
**Incomplete data outcome**	LOW	Outcomes relevant for the present meta-analysis mentioned in the full CSR were reported in the Journal article.
**Selective reporting**	UNCLEAR	No details on selective reporting.
**Other bias**	UNCLEAR	No details on other bias.
**Notes**	Author contacted but no reply.	

Jagannathan P et al [38]

ITEM	RATING	SUPPORT
**Random sequence generation**	LOW	Received medications and placebo in a random order, double-blind .
**Allocation concealment**	LOW	Information from the Journal article, Method of Assigning Subjects to Treatment Groups.
**Blinding participants/personnel**	UNCLEAR	No protocol/CSR available.
**Blinding assessor**	UNCLEAR	No protocol/CSR available.
**Incomplete data outcome**	LOW	Outcomes relevant for the present meta-analysis mentioned were reported in the Journal article.
**Selective reporting**	UNCLEAR	No protocol/CSR available.
**Other bias**	UNCLEAR	No protocol/CSR available.
**Notes**	First author contacted but not able to provide additional information.	

Jeronimo CMP et al [39]

ITEM	RATING	SUPPORT
**Random sequence generation**	LOW	Received medications and placebo in a random order, double-blind .
**Allocation concealment**	LOW	Information from full CSR, Method of Assigning Subjects to Treatment Groups.
**Blinding participants/personnel**	LOW	“Investigator and the patient were blinded to treatment. To maintain blinding, all investigational products were supplied.“ Information from full CSR, Blinding.
**Blinding assessor**	LOW	“Investigator and the patient were blinded to treatment. To maintain blinding, all investigational products were supplied.“ Information from full CSR, Blinding.
**Incomplete data outcome**	UNCLEAR	No details on incomplete data outcome.
**Selective reporting**	UNCLEAR	No details on selective reporting.
**Other bias**	UNCLEAR	No details on other bias.
**Notes**	Author contacted but no reply.	

Lenze EJ et al [40]

ITEM	RATING	SUPPORT

**Random sequence generation**	LOW	Received medications and placebo in a random order, double-blind .
**Allocation concealment**	LOW	Information from full CSR, Method of Assigning Subjects to Treatment Groups.
**Blinding participants/personnel**	LOW	“Investigator and the patient were blinded to treatment. To maintain blinding, all investigational products were supplied.“ Information from full CSR, Blinding.
**Blinding assessor**	LOW	“Investigator and the patient were blinded to treatment. To maintain blinding, all investigational products were supplied.“ Information from full CSR, Blinding.
**Incomplete data outcome**	LOW	Outcomes relevant for the present meta-analysis mentioned in the full CSR were reported in the Journal article.
**Selective reporting**	UNCLEAR	No details on selective reporting.
**Other bias**	UNCLEAR	No details on other bias.
**Notes**	Corresponding author contacted but not able to provide additional information.	

López-Medina E et al [41]

**ITEM**	**RATING**	**SUPPORT**
**Random sequence generation**	LOW	Received medications and placebo in a random order, double-blind .
**Allocation concealment**	LOW	Information from full CSR, Method of Assigning Subjects to Treatment Groups.
**Blinding participants/personnel**	LOW	“Investigator and the patient were blinded to treatment. To maintain blinding, all investigational products were supplied.“ Information from full CSR, Blinding.
**Blinding assessor**	LOW	“Investigator and the patient were blinded to treatment. To maintain blinding, all investigational products were supplied.“ Information from full CSR, Blinding.
**Incomplete data outcome**	UNCLEAR	No details on incomplete data outcome.
**Selective reporting**	UNCLEAR	No details on selective reporting.
**Other bias**	UNCLEAR	No details on other bias.
**Notes**	First author provided additional information.	

Marconi VC et al [42]

**ITEM**	**RATING**	**SUPPORT**
**Random sequence generation**	LOW	Received medications and placebo in a random order, double-blind .
**Allocation concealment**	LOW	Information from full CSR, Method of Assigning Subjects to Treatment Groups.
**Blinding participants/personnel**	LOW	“Investigator and the patient were blinded to treatment. To maintain blinding, all investigational products were supplied.“ Information from full CSR, Blinding.
**Blinding assessor**	LOW	“Investigator and the patient were blinded to treatment. To maintain blinding, all investigational products were supplied.“ Information from full CSR, Blinding.
**Incomplete data outcome**	LOW	Outcomes relevant for the present meta-analysis mentioned in the full CSR were reported in the Journal article.
**Selective reporting**	LOW	Outcomes pertinent to the present meta-analysis identified in the full CSR reported.
**Other bias**	UNCLEAR	No details on other bias.

Notes	Corresponding author contacted but no reply.

McCoy J et al [43]

ITEM	RATING	SUPPORT
Random sequence generation	LOW	Received medications and placebo in a random order, double-blind .
Allocation concealment	LOW	Information from full CSR, Method of Assigning Subjects to Treatment Groups.
Blinding participants/personnel	LOW	“Investigator and the patient were blinded to treatment. To maintain blinding, all investigational products were supplied.“ Information from full CSR, Blinding.
Blinding assessor	LOW	“Investigator and the patient were blinded to treatment. To maintain blinding, all investigational products were supplied.“ Information from full CSR, Blinding.
Incomplete data outcome	UNCLEAR	No details on incomplete data outcome.
Selective reporting	UNCLEAR	No details on selective reporting.
Other bias	UNCLEAR	No details on other bias.
Notes	First author provided additional information.	

Mohan A et al [44]

ITEM	RATING	SUPPORT
Random sequence generation	LOW	Received medications and placebo in a random order, double-blind .
Allocation concealment	LOW	Information from full CSR, Method of Assigning Subjects to Treatment Groups.
Blinding participants/personnel	LOW	“Investigator and the patient were blinded to treatment. To maintain blinding, all investigational products were supplied.“ Information from full CSR, Blinding.
Blinding assessor	LOW	“Investigator and the patient were blinded to treatment. To maintain blinding, all investigational products were supplied.“ Information from full CSR, Blinding.
Incomplete data outcome	UNCLEAR	No details on incomplete data outcome.
Selective reporting	UNCLEAR	No details on selective reporting.
Other bias	UNCLEAR	No details on other bias.
Notes	First author contacted but not able to provide additional information.	

Monk PD et al [45]

ITEM	RATING	SUPPORT
Random sequence generation	LOW	Received medications and placebo in a random order, double-blind .
Allocation concealment	LOW	Information from full CSR, Method of Assigning Subjects to Treatment Groups.
Blinding participants/personnel	LOW	“Investigator and the patient were blinded to treatment. To maintain blinding, all investigational products were supplied.“ Information from full CSR, Blinding.
Blinding assessor	LOW	“Investigator and the patient were blinded to treatment. To maintain blinding, all investigational products were supplied.“ Information from full CSR, Blinding.
Incomplete data outcome	LOW	Outcomes relevant for the present meta-analysis mentioned in the full CSR were reported in the Journal article.

**Selective reporting**	UNCLEAR	No details on selective reporting.
**Other bias**	UNCLEAR	No details on other bias.
**Notes**	First author contacted but not able to provide additional information.	

Omrani AS et al [46]

ITEM	RATING	SUPPORT
**Random sequence generation**	LOW	Received medications and placebo in a random order, double-blind .
**Allocation concealment**	LOW	Information from full article, Method of Assigning Subjects to Treatment Groups.
**Blinding participants/personnel**	HIGH	No details on who was blinded and how blinding was preserved.
**Blinding assessor**	UNCLEAR	No protocol/CSR available.
**Incomplete data outcome**	UNCLEAR	No protocol/CSR available.
**Selective reporting**	UNCLEAR	No protocol/CSR available.
**Other bias**	HIGH	No protocol/CSR available.
**Notes**	Corresponding author not able to provide additional details.	

Patel O et al [47]

ITEM	RATING	SUPPORT
**Random sequence generation**	LOW	Received medications and placebo in a random order, double-blind .
**Allocation concealment**	LOW	Information from full CSR, Method of Assigning Subjects to Treatment Groups.
**Blinding participants/personnel**	LOW	"Investigator and the patient were blinded to treatment. To maintain blinding, all investigational products were supplied." Information from full CSR, Blinding.
**Blinding assessor**	UNCLEAR	Not specified.
**Incomplete data outcome**	LOW	Outcomes relevant for the present meta-analysis mentioned in the full CSR were reported in the Journal article.
**Selective reporting**	LOW	All outcomes of relevance reported in published journal article.
**Other bias**	UNCLEAR	Not specified.
**Notes**	First author provided additional information.	

Puskarich MA et al [48]

ITEM	RATING	SUPPORT
**Random sequence generation**	LOW	Received medications and placebo in a random order, double-blind .
**Allocation concealment**	LOW	Information from full CSR, Method of Assigning Subjects to Treatment Groups.
**Blinding participants/personnel**	LOW	"Investigator and the patient were blinded to treatment. To maintain blinding, all investigational products were supplied." Information from full CSR, Blinding.
**Blinding assessor**	LOW	"Investigator and the patient were blinded to treatment. To maintain blinding, all investigational products were supplied." Information from full CSR, Blinding.
**Incomplete data outcome**	LOW	Outcomes relevant for the present meta-analysis mentioned in the full CSR were reported in the Journal article.

**Selective reporting**	LOW	All outcomes of relevance reported in published journal article.
**Other bias**	UNCLEAR	Not specified.
**Notes**	First author contacted but not able to provide additional information.	

Rastogi A et al [49]

**ITEM**	**RATING**	**SUPPORT**
**Random sequence generation**	LOW	Received medications and placebo in a random order, double-blind .
**Allocation concealment**	LOW	Information from journal article, Method of Assigning Subjects to Treatment Groups.
**Blinding participants/personnel**	UNCLEAR	No protocol/CSR available.
**Blinding assessor**	UNCLEAR	No protocol/CSR available.
**Incomplete data outcome**	UNCLEAR	No protocol/CSR available.
**Selective reporting**	LOW	All outcomes of relevance reported in published journal article.
**Other bias**	UNCLEAR	No protocol/CSR available.
**Notes**	First author unable to provide additional details.	

Ravikirti et al [50]

**ITEM**	**RATING**	**SUPPORT**
**Random sequence generation**	LOW	Received medications and placebo in a random order, double-blind .
**Allocation concealment**	LOW	Information from full article, Method of Assigning Subjects to Treatment Groups.
**Blinding participants/personnel**	UNCLEAR	No protocol/CSR available.
**Blinding assessor**	UNCLEAR	No protocol/CSR available.
**Incomplete data outcome**	UNCLEAR	No protocol/CSR available.
**Selective reporting**	LOW	All outcomes of relevance reported in published journal article.
**Other bias**	UNCLEAR	No protocol/CSR available.
**Notes**	First author contacted but not able to provide additional information.	

Reis G et al [51]

**ITEM**	**RATING**	**SUPPORT**
**Random sequence generation**	LOW	Received medications and placebo in a random order, double-blind .
**Allocation concealment**	LOW	Information from full CSR, Method of Assigning Subjects to Treatment Groups.
**Blinding participants/personnel**	HIGH	No details on who was blinded and how blinding was preserved.
**Blinding assessor**	HIGH	No details on who was blinded and how blinding was preserved.
**Incomplete data outcome**	LOW	Outcomes relevant for the present meta-analysis mentioned in the full CSR were reported in the Journal article.
**Selective reporting**	UNCLEAR	Not specified.
**Other bias**	LOW	All reported in published papers.

**Notes**	First author unable to provide additional details.

Rocco PRM et al [52]

ITEM	RATING	SUPPORT
**Random sequence generation**	LOW	Received medications and placebo in a random order, double-blind .
**Allocation concealment**	LOW	Information from the full article, Method of Assigning Subjects to Treatment Groups.
**Blinding participants/personnel**	LOW	"Investigator and the patient were blinded to treatment. To maintain blinding, all investigational products were supplied." Information from the full article, Blinding.
**Blinding assessor**	UNCLEAR	No protocol/CSR available.
**Incomplete data outcome**	UNCLEAR	No protocol/CSR available.
**Selective reporting**	LOW	All outcomes of relevance reported in published journal article.
**Other bias**	LOW	All reported in published papers.
**Notes**	First author unable to provide additional details.	

Salama C et al [53]

ITEM	RATING	SUPPORT
**Random sequence generation**	LOW	Received medications and placebo in a random order, double-blind .
**Allocation concealment**	LOW	Information from the full article, Method of Assigning Subjects to Treatment Groups.
**Blinding participants/personnel**	LOW	"Investigator and the patient were blinded to treatment. To maintain blinding, all investigational products were supplied." Information from the full article, Blinding.
**Blinding assessor**	LOW	"Investigator and the patient were blinded to treatment. To maintain blinding, all investigational products were supplied." Information from the full article, Blinding.
**Incomplete data outcome**	UNCLEAR	No protocol/CSR available.
**Selective reporting**	UNCLEAR	No protocol/CSR available.
**Other bias**	UNCLEAR	No protocol/CSR available.
**Notes**	First author unable to provide additional details.	

Silva M et al [54]

ITEM	RATING	SUPPORT
**Random sequence generation**	LOW	Received medications and placebo in a random order, double-blind .
**Allocation concealment**	LOW	Information from the full article, Method of Assigning Subjects to Treatment Groups.
**Blinding participants/personnel**	LOW	"Investigator and the patient were blinded to treatment. To maintain blinding, all investigational products were supplied." Information from the full article, Blinding.
**Blinding assessor**	LOW	"Investigator and the patient were blinded to treatment. To maintain blinding, all investigational products were supplied." Information from the full article, Blinding.
**Incomplete data outcome**	UNCLEAR	No protocol/CSR available.
**Selective reporting**	UNCLEAR	No protocol/CSR available.
**Other bias**	UNCLEAR	No protocol/CSR available.

**Notes**	First author contacted but not able to provide additional information.

Sivapalan P et al [55]

ITEM	RATING	SUPPORT
**Random sequence generation**	LOW	Received medications and placebo in a random order, double-blind .
**Allocation concealment**	LOW	Information from full CSR, Method of Assigning Subjects to Treatment Groups.
**Blinding participants/personnel**	LOW	“Investigator and the patient were blinded to treatment. To maintain blinding, all investigational products were supplied.“ Information from full CSR, Blinding.
**Blinding assessor**	LOW	“Investigator and the patient were blinded to treatment. To maintain blinding, all investigational products were supplied.“ Information from full CSR, Blinding.
**Incomplete data outcome**	UNCLEAR	Not specified.
**Selective reporting**	HIGH	Unbalanced reasons for selective reporting.
**Other bias**	LOW	No protocol/CSR available.
**Notes**	First author contacted but not able to provide additional information.	

Skipper CP et al [56]

ITEM	RATING	SUPPORT
**Random sequence generation**	LOW	Received medications and placebo in a random order, double-blind .
**Allocation concealment**	LOW	Information from full CSR, Method of Assigning Subjects to Treatment Groups.
**Blinding participants/personnel**	LOW	“Investigator and the patient were blinded to treatment. To maintain blinding, all investigational products were supplied.“ Information from full CSR, Blinding.
**Blinding assessor**	LOW	“Investigator and the patient were blinded to treatment. To maintain blinding, all investigational products were supplied.“ Information from full CSR, Blinding.
**Incomplete data outcome**	LOW	Outcomes relevant for the present meta-analysis mentioned in the full CSR were reported in the Journal article.
**Selective reporting**	UNCLEAR	Not specified.
**Other bias**	LOW	All information listed in clinialtrial.gov was reported in the journal article.
**Notes**	Authors provided additional information.	

Stone JH et al [57]

ITEM	RATING	SUPPORT
**Random sequence generation**	LOW	Received medications and placebo in a random order, double-blind .
**Allocation concealment**	LOW	Information from full CSR, Method of Assigning Subjects to Treatment Groups.
**Blinding participants/personnel**	LOW	“Investigator and the patient were blinded to treatment. To maintain blinding, all investigational products were supplied.“ Information from full CSR, Blinding.
**Blinding assessor**	LOW	“Investigator and the patient were blinded to treatment. To maintain blinding, all investigational products were supplied.“ Information from full CSR, Blinding.
**Incomplete data outcome**	UNCLEAR	Not specified.

**Selective reporting**	LOW	All outcomes listed in clinialtrial.gov were reported in the journal article (which included additional outcomes)
**Other bias**	UNCLEAR	Not specified.
**Notes**	Authors provided additional information.	

Tardif JC et al [58]

**ITEM**	**RATING**	**SUPPORT**
**Random sequence generation**	LOW	Received medications and placebo in a random order, double-blind .
**Allocation concealment**	LOW	Information from full CSR, Method of Assigning Subjects to Treatment Groups.
**Blinding participants/personnel**	LOW	“Investigator and the patient were blinded to treatment. To maintain blinding, all investigational products were supplied.“ Information from full CSR, Blinding.
**Blinding assessor**	LOW	“Investigator and the patient were blinded to treatment. To maintain blinding, all investigational products were supplied.“ Information from full CSR, Blinding.
**Incomplete data outcome**	LOW	Outcomes relevant for the present meta-analysis mentioned in the full CSR were reported in the Journal article.
**Selective reporting**	LOW	All outcomes listed in clinialtrial.gov were reported in the journal article (which included additional outcomes)
**Other bias**	UNCLEAR	Not specified.
**Notes**	Authors provided additional information.	

Tornling G et al [59]

**ITEM**	**RATING**	**SUPPORT**
**Random sequence generation**	LOW	Received medications and placebo in a random order, double-blind .
**Allocation concealment**	LOW	Information from full CSR, Method of Assigning Subjects to Treatment Groups.
**Blinding participants/personnel**	LOW	“Investigator and the patient were blinded to treatment. To maintain blinding, all investigational products were supplied.“ Information from full CSR, Blinding.
**Blinding assessor**	UNCLEAR	Not specified.
**Incomplete data outcome**	LOW	Outcomes relevant for the present meta-analysis mentioned in the full CSR were reported in the Journal article.
**Selective reporting**	UNCLEAR	Not specified.
**Other bias**	LOW	All information listed in clinialtrial.gov was reported in the journal article.
**Notes**	First author contacted but not able to provide additional information.	

Ulrich RJ et al [60]

**ITEM**	**RATING**	**SUPPORT**
**Random sequence generation**	LOW	Received medications and placebo in a random order, double-blind .
**Allocation concealment**	LOW	Information from full CSR, Method of Assigning Subjects to Treatment Groups.
**Blinding participants/personnel**	LOW	“Investigator and the patient were blinded to treatment. To maintain blinding, all investigational products were supplied.“ Information from full CSR, Blinding.

**Blinding assessor**	LOW	Throughout, a pharmacy prepared, distributed and tracked all the drugs, separately from raters and subjects in order to maintain double-blind conditions.
**Incomplete data outcome**	UNCLEAR	Not specified.
**Selective reporting**	LOW	All outcomes listed in clinialtrial.gov were reported in the journal article (which included additional outcomes)
**Other bias**	UNCLEAR	Not specified.
**Notes**	Authors provided additional information.	

Vallejos J et al [61]

ITEM	RATING	SUPPORT
**Random sequence generation**	LOW	Received medications and placebo in a random order, double-blind .
**Allocation concealment**	LOW	Information from full CSR, Method of Assigning Subjects to Treatment Groups.
**Blinding participants/personnel**	LOW	“Investigator and the patient were blinded to treatment. To maintain blinding, all investigational products were supplied.“ Information from full CSR, Blinding.
**Blinding assessor**	LOW	Throughout, a pharmacy prepared, distributed and tracked all the drugs, separately from raters and subjects in order to maintain double-blind conditions.
**Incomplete data outcome**	UNCLEAR	Not specified.
**Selective reporting**	UNCLEAR	Not specified.
**Other bias**	LOW	All information listed in clinialtrial.gov was reported in the journal article.
**Notes**	Authors provided additional information.	

Wang Y et al [62]

ITEM	RATING	SUPPORT
**Random sequence generation**	LOW	Received medications and placebo in a random order, double-blind .
**Allocation concealment**	LOW	Information from full CSR, Method of Assigning Subjects to Treatment Groups.
**Blinding participants/personnel**	LOW	“Investigator and the patient were blinded to treatment. To maintain blinding, all investigational products were supplied.“ Information from full CSR, Blinding.
**Blinding assessor**	LOW	Throughout, a pharmacy prepared, distributed and tracked all the drugs, separately from raters and subjects in order to maintain double-blind conditions.
**Incomplete data outcome**	LOW	Outcomes relevant for the present meta-analysis mentioned in the full CSR were reported in the Journal article.
**Selective reporting**	LOW	All outcomes listed in clinialtrial.gov were reported in the journal article (which included additional outcomes)
**Other bias**	UNCLEAR	Not specified.
**Notes**	First author contacted but not able to provide additional information.	

Weinreich DM et al [63]

ITEM	RATING	SUPPORT
**Random sequence generation**	LOW	Received medications and placebo in a random order, double-blind .
**Allocation concealment**	LOW	Information from full CSR, Method of Assigning Subjects to Treatment Groups.

**Blinding participants/personnel**	LOW	"Investigator and the patient were blinded to treatment. To maintain blinding, all investigational products were supplied." Information from full CSR, Blinding.
**Blinding assessor**	LOW	Throughout, a pharmacy prepared, distributed and tracked all the drugs, separately from raters and subjects in order to maintain double-blind conditions.
**Incomplete data outcome**	LOW	Outcomes relevant for the present meta-analysis mentioned in the full CSR were reported in the Journal article.
**Selective reporting**	UNCLEAR	Not specified.
**Other bias**	UNCLEAR	Not specified.
**Notes**	Authors not able to provide additional information.	

CaoY et al [64]

ITEM	RATING	SUPPORT
**Random sequence generation**	LOW	Received medications and placebo in a random order, double-blind .
**Allocation concealment**	LOW	Information from full CSR, Method of Assigning Subjects to Treatment Groups.
**Blinding participants/personnel**	HIGH	No details on who was blinded and how blinding was preserved.
**Blinding assessor**	HIGH	No details on who was blinded and how blinding was preserved.
**Incomplete data outcome**	UNCLEAR	Not specified.
**Selective reporting**	LOW	All outcomes listed in clinialtrial.gov were reported in the journal article (which included additional outcomes)
**Other bias**	UNCLEAR	Not specified.
**Notes**	First author contacted but not able to provide additional information.	

Cremer PC et al [65]

ITEM	RATING	SUPPORT
**Random sequence generation**	LOW	Received medications and placebo in a random order, double-blind .
**Allocation concealment**	UNCLEAR	Not specified.
**Blinding participants/personnel**	LOW	"Investigator and the patient were blinded to treatment. To maintain blinding, all investigational products were supplied." Information from full CSR, Blinding.
**Blinding assessor**	LOW	Throughout, a pharmacy prepared, distributed and tracked all the drugs, separately from raters and subjects in order to maintain double-blind conditions.
**Incomplete data outcome**	LOW	Outcomes relevant for the present meta-analysis mentioned in the full CSR were reported in the Journal article.
**Selective reporting**	LOW	All outcomes listed in clinialtrial.gov were reported in the journal article (which included additional outcomes)
**Other bias**	UNCLEAR	Not specified.
**Notes**	Authors not able to provide additional information.	

de Alencar JCG et al [66]

ITEM	RATING	SUPPORT
**Random sequence generation**	LOW	Received medications and placebo in a random order, double-blind .

**Allocation concealment**	LOW	Information from full CSR, Method of Assigning Subjects to Treatment Groups.
**Blinding participants/personnel**	LOW	“Investigator and the patient were blinded to treatment. To maintain blinding, all investigational products were supplied.“ Information from full CSR, Blinding.
**Blinding assessor**	LOW	Throughout, a pharmacy prepared, distributed and tracked all the drugs, separately from raters and subjects in order to maintain double-blind conditions.
**Incomplete data outcome**	LOW	Outcomes relevant for the present meta-analysis mentioned in the full CSR were reported in the Journal article.
**Selective reporting**	UNCLEAR	Not specified.
**Other bias**	UNCLEAR	Not specified.
**Notes**	First author contacted but not able to provide additional information.	

Dequin PF et al [67]

ITEM	RATING	SUPPORT
**Random sequence generation**	LOW	Received medications and placebo in a random order, double-blind .
**Allocation concealment**	LOW	Information from full CSR, Method of Assigning Subjects to Treatment Groups.
**Blinding participants/personnel**	LOW	Throughout, a pharmacy prepared, distributed and tracked all the drugs, separately from raters and subjects in order to maintain double-blind conditions.
**Blinding assessor**	LOW	“Investigator and the patient were blinded to treatment. To maintain blinding, all investigational products were supplied.“ Information from full CSR, Blinding.
**Incomplete data outcome**	UNCLEAR	Not specified.
**Selective reporting**	UNCLEAR	Not specified.
**Other bias**	UNCLEAR	Not specified.
**Notes**	Manufacturer provided additional information.	

Gharebaghi N et al [68]

ITEM	RATING	SUPPORT
**Random sequence generation**	LOW	Received medications and placebo in a random order, double-blind .
**Allocation concealment**	LOW	Information from full CSR, Method of Assigning Subjects to Treatment Groups.
**Blinding participants/personnel**	LOW	Throughout, a pharmacy prepared, distributed and tracked all the drugs, separately from raters and subjects in order to maintain double-blind conditions.
**Blinding assessor**	LOW	“Investigator and the patient were blinded to treatment. To maintain blinding, all investigational products were supplied.“ Information from full CSR, Blinding.
**Incomplete data outcome**	UNCLEAR	Not specified.
**Selective reporting**	LOW	All outcomes listed in clinialtrial.gov were reported in the journal article (which included additional outcomes)
**Other bias**	UNCLEAR	Not specified.
**Notes**	First author contacted but not able to provide additional information.	

Lescure FX et al [69]

ITEM	RATING	SUPPORT
**Random sequence generation**	LOW	Received medications and placebo in a random order, double-blind .
**Allocation concealment**	LOW	Information from full CSR, Method of Assigning Subjects to Treatment Groups.
**Blinding participants/personnel**	LOW	“Investigator and the patient were blinded to treatment. To maintain blinding, all investigational products were supplied.“ Information from full CSR, Blinding.
**Blinding assessor**	LOW	“Investigator and the patient were blinded to treatment. To maintain blinding, all investigational products were supplied.“ Information from full CSR, Blinding.
**Incomplete data outcome**	LOW	Outcomes relevant for the present meta-analysis mentioned in the full CSR were reported in the Journal article.
**Selective reporting**	LOW	All outcomes listed in clinialtrial.gov were reported in the journal article (which included additional outcomes)
**Other bias**	UNCLEAR	Not specified.
**Notes**	Authors not able to provide additional information.	

Libster R et al [70]

ITEM	RATING	SUPPORT
**Random sequence generation**	LOW	Received medications and placebo in a random order, double-blind .
**Allocation concealment**	LOW	Information from full CSR, Method of Assigning Subjects to Treatment Groups.
**Blinding participants/personnel**	LOW	“Investigator and the patient were blinded to treatment. To maintain blinding, all investigational products were supplied.“ Information from full CSR, Blinding.
**Blinding assessor**	UNCLEAR	Not specified.
**Incomplete data outcome**	LOW	Outcomes relevant for the present meta-analysis mentioned in the full CSR were reported in the Journal article.
**Selective reporting**	UNCLEAR	Not specified.
**Other bias**	UNCLEAR	Not specified.
**Notes**	Contacted author but not valid e-mail; no other e-mail addresses found.	

Munch MW et al [71]

ITEM	RATING	SUPPORT
**Random sequence generation**	LOW	Received medications and placebo in a random order, double-blind .
**Allocation concealment**	LOW	Information from full CSR, Method of Assigning Subjects to Treatment Groups.
**Blinding participants/personnel**	LOW	“Investigator and the patient were blinded to treatment. To maintain blinding, all investigational products were supplied.“ Information from full CSR, Blinding.
**Blinding assessor**	LOW	“Investigator and the patient were blinded to treatment. To maintain blinding, all investigational products were supplied.“ Information from full CSR, Blinding.
**Incomplete data outcome**	LOW	Outcomes relevant for the present meta-analysis mentioned in the full CSR were reported in the Journal article.
**Selective reporting**	UNCLEAR	Not specified.

**Other bias**	LOW	All information listed in clinialtrial.gov was reported in the journal article.
**Notes**	Contacted author but not valid e-mail; no other e-mail addresses found.	

Rosas IO et al [72]

ITEM	RATING	SUPPORT
**Random sequence generation**	LOW	Received medications and placebo in a random order, double-blind .
**Allocation concealment**	LOW	Information from full CSR, Method of Assigning Subjects to Treatment Groups.
**Blinding participants/personnel**	LOW	“Investigator and the patient were blinded to treatment. To maintain blinding, all investigational products were supplied.“ Information from full CSR, Blinding.
**Blinding assessor**	LOW	“Investigator and the patient were blinded to treatment. To maintain blinding, all investigational products were supplied.“ Information from full CSR, Blinding.
**Incomplete data outcome**	LOW	Outcomes relevant for the present meta-analysis mentioned in the full CSR were reported in the Journal article.
**Selective reporting**	LOW	Outcomes listed in short CSR reported in the journal article.
**Other bias**	UNCLEAR	Not specified.
**Notes**	E-mail address for corresponding author not valid; no other email addresses found.	

Sehgal IS et al [73]

ITEM	RATING	SUPPORT
**Random sequence generation**	LOW	Received medications and placebo in a random order, double-blind .
**Allocation concealment**	LOW	Information from full CSR, Method of Assigning Subjects to Treatment Groups.
**Blinding participants/personnel**	LOW	“Investigator and the patient were blinded to treatment. To maintain blinding, all investigational products were supplied.“ Information from full CSR, Blinding.
**Blinding assessor**	UNCLEAR	Not specified.
**Incomplete data outcome**	LOW	Outcomes relevant for the present meta-analysis mentioned in the full CSR were reported in the Journal article.
**Selective reporting**	UNCLEAR	Not specified.
**Other bias**	UNCLEAR	Not specified.
**Notes**	First author contacted but not able to provide additional information.	

Shi L et al [74]

ITEM	RATING	SUPPORT
**Random sequence generation**	LOW	Received medications and placebo in a random order, double-blind .
**Allocation concealment**	LOW	Information from full CSR, Method of Assigning Subjects to Treatment Groups.
**Blinding participants/personnel**	LOW	“Investigator and the patient were blinded to treatment. To maintain blinding, all investigational products were supplied.“ Information from full CSR, Blinding.
**Blinding assessor**	UNCLEAR	Not specified.

**Incomplete data outcome**	LOW	Outcomes relevant for the present meta-analysis mentioned in the full CSR were reported in the Journal article.
**Selective reporting**	LOW	Outcomes listed in short CSR reported in the journal article.
**Other bias**	UNCLEAR	Not specified.
**Notes**	Manufacturer provided additional information.	

Simonovich VA et al [75]

ITEM	RATING	SUPPORT
**Random sequence generation**	LOW	Received medications and placebo in a random order, double-blind .
**Allocation concealment**	LOW	Information from full CSR, Method of Assigning Subjects to Treatment Groups.
**Blinding participants/personnel**	UNCLEAR	Not specified.
**Blinding assessor**	UNCLEAR	Not specified.
**Incomplete data outcome**	UNCLEAR	Not specified.
**Selective reporting**	LOW	Outcomes listed in short CSR reported in the journal article.
**Other bias**	UNCLEAR	Not specified.
**Notes**	First author contacted but not able to provide additional information.	

Sivapalasingam S et al [76]

ITEM	RATING	SUPPORT
**Random sequence generation**	LOW	Received medications and placebo in a random order, double-blind .
**Allocation concealment**	LOW	Information from full CSR, Method of Assigning Subjects to Treatment Groups.
**Blinding participants/personnel**	LOW	"Investigator and the patient were blinded to treatment. To maintain blinding, all investigational products were supplied." Information from full CSR, Blinding.
**Blinding assessor**	LOW	"Investigator and the patient were blinded to treatment. To maintain blinding, all investigational products were supplied." Information from full CSR, Blinding.
**Incomplete data outcome**	LOW	Outcomes relevant for the present meta-analysis mentioned in the full CSR were reported in the Journal article.
**Selective reporting**	UNCLEAR	Not specified.
**Other bias**	UNCLEAR	Not specified.
**Notes**	Authors not able to provide additional information.	

Temesgen Z et al [77]

ITEM	RATING	SUPPORT
**Random sequence generation**	LOW	Received medications and placebo in a random order, double-blind .
**Allocation concealment**	LOW	Information from full CSR, Method of Assigning Subjects to Treatment Groups.
**Blinding participants/personnel**	LOW	"Investigator and the patient were blinded to treatment. To maintain blinding, all investigational products were supplied." Information from full CSR, Blinding.

**Blinding assessor**	LOW	“Investigator and the patient were blinded to treatment. To maintain blinding, all investigational products were supplied.“ Information from full CSR, Blinding.
**Incomplete data outcome**	LOW	Outcomes relevant for the present meta-analysis mentioned in the full CSR were reported in the Journal article.
**Selective reporting**	UNCLEAR	Not specified.
**Other bias**	UNCLEAR	Not specified.
**Notes**	First author contacted but not able to provide additional information.	

Zhong M et al [78]

**ITEM**	**RATING**	**SUPPORT**
**Random sequence generation**	LOW	Received medications and placebo in a random order, double-blind .
**Allocation concealment**	LOW	Information from full CSR, Method of Assigning Subjects to Treatment Groups.
**Blinding participants/personnel**	UNCLEAR	Not specified.
**Blinding assessor**	UNCLEAR	Not specified.
**Incomplete data outcome**	LOW	Outcomes relevant for the present meta-analysis mentioned in the full CSR were reported in the Journal article.
**Selective reporting**	LOW	Outcomes listed in short CSR reported in the journal article.
**Other bias**	UNCLEAR	Not specified.
**Notes**	Authors not able to provide additional information.	
