# Supplementary material for: Efficacy and safety of current medications for treating severe and non-severe COVID-19 patients: an updated network meta-analysis of randomized placebo-controlled trials
Source: Aging (Albany NY). 2021 Sep 16;13(18):21866–902. doi: 10.18632/aging.203522 (PMC8507270; doi:10.18632/aging.203522)
Supplement: Supplementary Tables 3-9 [file aging-13-203522-s004.pdf]

SUPPLEMENTARY TABLES

Supplementary Table 3. Risk of bias table of included studies.

Study ID (reference)	Low risk of bias (%)	High risk of bias (%)	Unclear risk of bias (%)
ACTIV-3/TICO LY-CoV555 Study Group [18]	85.71	0	14.29
Ahmed S et al. [19]	71.43	0	28.57
Ahmed S et al. [19]	71.43	0	28.57
Aman J et al. [20]	71.43	0	28.57
Biber A et al. [21]	71.43	0	28.57
Blum VF et al. [22]	71.43	0	28.57
Cadegiani FA et al. (a) [23]	85.71	0	14.29
Cadegiani FA et al. (b) [24]	71.43	0	28.57
Caricchio R et al. [25]	71.43	0	28.57
Chaccour C et al. [26]	57.14	0	42.86
Chen J et al. [27]	57.14	0	42.86
Devpura G et al. [28]	71.43	0	28.57
Dubee V et al. [29]	85.71	0	14.29
Eom JS et al. [30]	71.43	0	28.57
Feld JJ et al. [31]	85.71	0	14.29
Gonzalez-Ochoa AG et al. [32]	57.14	28.57	14.29
Gottlieb RL et al. [33]	85.71	0	14.29
Gunst GD et al. [34]	85.71	0	14.29
Gupta A et al. [35]	85.71	0	14.29
Gutierrez-Castrellon P et al. [36]	71.43	0	28.57
Humeniuk R et al. [37]	71.43	0	28.57
Jagannathan P et al. [38]	42.86	0	57.14
Jeronimo CMP et al. [39]	57.14	0	42.86
Lenze EJ et al. [40]	71.43	0	28.57
López-Medina E et al. [41]	57.14	0	42.86
Marconi VC et al. [42]	85.71	0	14.29
McCoy J et al. [43]	57.14	0	42.86
Mohan A et al. [44]	57.14	0	42.86
Monk PD et al. [45]	71.43	0	28.57
Omrani AS et al. [46]	28.57	28.57	42.86
Patel O et al. [47]	71.43	0	28.57
Puskarich MA et al. [48]	85.71	0	14.29
Rastogi A et al. [49]	42.86	0	57.14
Ravikirti et al. [50]	42.86	0	57.14
Reis G et al. [51]	57.14	28.57	14.29
Rocco PRM et al. [52]	71.43	0	28.57
Salama C et al. [53]	57.14	0	42.86
Silva M et al. [54]	57.14	0	42.86

Sivapalan P et al. [55]	71.43	14.28	14.29
Skipper CP et al. [56]	85.71	0	14.29
Stone JH et al. [57]	71.43	0	28.57
Tardif JC et al. [58]	85.71	0	14.29
Tornling G et al. [59]	71.43	0	28.57
Ulrich RJ et al. [60]	71.43	0	28.57
Vallejos J et al. [61]	71.43	0	28.57
Wang Y et al. [62]	85.71	0	14.29
Weinreich DM et al. [63]	71.43	0	28.57
CaoY et al. [64]	42.86	28.57	28.57
Cremer PC et al. [65]	71.43	0	28.57
de Alencar JCG et al. [66]	71.43	0	28.57
Dequin PF et al. [67]	57.14	0	42.86
Gharebaghi N et al. [68]	71.43	0	28.57
Lescure FX et al. [69]	85.71	0	14.29
Libster R et al. [70]	57.14	0	42.86
Munch MW et al. [71]	85.71	0	14.29
Rosas IO et al. [72]	85.71	0	14.29
Sehgal IS et al. [73]	57.14	0	42.86
Shi L et al. [74]	71.43	0	28.57
Simonovich VA et al. [75]	42.86	0	57.14
Sivapalasingam S et al. [76]	71.43	0	28.57
Temesgen Z et al. [77]	71.43	0	28.57
Zhong M et al. [78]	57.14	0	42.86

Supplementary Table 4. Included studies of network meta-analysis for the ratio of virological cure in non-severe patients with COVID-19.

Study (reference)	Intervention and control groups	Events	N	Ratio of virological cure (%)
Ahmed S et al. 2021 [19]	Ivermectin	17	22	77.27
Ahmed S et al. 2021 [19]	Ivermectin/doxycycline	14	23	60.87
Ahmed S et al. 2021 [19]	Placebo	9	23	39.13
Biber A et al. 2021 [21]	Ivermectin	39	47	82.98
Biber A et al. 2021 [21]	Placebo	25	42	59.52
Cadegiani FA et al. 2021 [23]	Proxalutamide	140	171	81.87
Cadegiani FA et al. 2021 [23]	Placebo	20	65	30.77
Chaccour C et al. 2021 [26]	Ivermectin	1	12	8.33
Chaccour C et al. 2021 [26]	Placebo	0.5	12	4.17
Chen J et al. 2020 [27]	Arbidol	19	23	82.61
Chen J et al. 2020 [27]	Placebo	27	35	77.14
Devpura G et al. 2021 [28]	Ayurvedic	45	45	100.00
Devpura G et al. 2021 [28]	Placebo	30	50	60.00
Dubee V et al. 2021 [29]	HCQ	39	91	42.86
Dubee V et al. 2021 [29]	Placebo	36	83	43.37
Eom JS et al. 2021 [30]	LCP	93	101	92.08
Eom JS et al. 2021 [30]	HCP	90	103	87.38
Eom JS et al. 2021 [30]	CPC	183	204	89.71
Eom JS et al. 2021 [30]	Placebo	86	103	83.50
Feld JJ et al. 2021 [31]	PL	24	30	80.00
Feld JJ et al. 2021 [31]	Placebo	19	30	63.33
Gottlieb RL et al. 2021 [33]	LDB	41	85	48.24
Gottlieb RL et al. 2021 [33]	MDB	43	93	46.24
Gottlieb RL et al. 2021 [33]	HDB	37	86	43.02
Gottlieb RL et al. 2021 [33]	MDB/etesevimab	40	82	48.78
Gottlieb RL et al. 2021 [33]	Placebo	56	122	45.90
Jeronimo CMP et al. 2021 [39]	Methylprednisolone	91	161	56.52
Jeronimo CMP et al. 2021 [39]	Placebo	88	157	56.05
Mohan A et al. 2020 [44]	Ivermectin	16	36	44.44
Mohan A et al. 2020 [44]	Placebo	14	45	31.11
Mohan A et al. 2020 [44]	LDI	13	36	36.11
Omrani AS et al. 2020 [46]	HCQ/AZM	30	149	20.13
Omrani AS et al. 2020 [46]	HCQ	42	146	28.77
Omrani AS et al. 2020 [46]	Placebo	45	143	31.47
Rastogi A et al. 2020 [49]	HDVD	10	16	62.50
Rastogi A et al. 2020 [49]	Placebo	5	24	20.83
Ravikirti et al. 2021 [50]	Ivermectin	13	55	23.64
Ravikirti et al. 2021 [50]	Placebo	18	57	31.58
Rocco PRM et al. 2020 [52]	Nitazoxanide	58	194	29.90
Rocco PRM et al. 2020 [52]	Placebo	36	198	18.18
Silva M et al. 2021 [54]	Nitazoxanide	14	23	60.87
Silva M et al. 2021 [54]	Placebo	7	13	53.85
Ulrich RJ et al. 2020 [60]	HCQ	8	67	11.94
Ulrich RJ et al. 2020 [60]	Placebo	10	61	16.39

Vallejos J et al. 2021 [61]	Ivermectin	212	250	84.80
Vallejos J et al. 2021 [61]	Placebo	221	251	88.05
Wang Y et al. 2020 [62]	Remdesivir	99	131	75.57
Wang Y et al. 2020 [62]	Placebo	54	65	83.08
Weinreich DM et al. 2021 [63]	REGN-COV2	80	182	43.96
Weinreich DM et al. 2021 [63]	Placebo	33	93	35.48

Abbreviations: COVID-19: coronavirus disease 2019; PL: peginterferon lambda; LDI: low dosage ivermectin; HDVD: high-dose vitamin D; LCP: low dosage CT-P59; HCP: high dosage CT-P59; CPC: CT-P59 combined; HCQ: hydroxychloroquine; AZM: azithromycin; LDB: low dosage bamlanivimab; MDB: moderate dosage bamlanivimab; HDB: high dosage bamlanivimab.

Supplementary Table 5. Included studies of network meta-analysis for all-cause mortality in non-severe patients with COVID-19.

Study (reference)	Intervention and control groups	Events	N	All-cause mortality (%)
ACTIV-3/TICO LY-CoV555 Study Group 2021 [18]	LY-CoV555	9	163	5.52
ACTIV-3/TICO LY-CoV555 Study Group 2021 [18]	Placebo	5	151	3.31
Aman J et al. 2021 [20]	Imatinib	15	197	7.61
Aman J et al. 2021 [20]	Placebo	27	188	14.36
Blum VF et al. 2021 [22]	Nitazoxanide	2	25	8.00
Blum VF et al. 2021 [22]	Placebo	6	25	24.00
Cadegiani FA et al. 2021 [24]	Proxalutamide	35	317	11.04
Cadegiani FA et al. 2021 [24]	Placebo	162	328	49.39
Caricchio R et al. 2021 [25]	Canakinumab	11	223	4.93
Caricchio R et al. 2021 [25]	Placebo	16	222	7.21
Dubee V et al. 2021 [29]	HCQ	6	124	4.84
Dubee V et al. 2021 [29]	Placebo	11	123	8.94
Eom JS et al. 2021 [30]	LCP	0	95	87.37
Eom JS et al. 2021 [30]	HCP	0	92	85.87
Eom JS et al. 2021 [30]	CPC	0	187	86.63
Eom JS et al. 2021 [30]	Placebo	0	98	71.43
Gonzalez-Ochoa AG et al. 2021 [32]	Sulodexide	3	124	2.42
Gonzalez-Ochoa AG et al. 2021 [32]	Placebo	7	119	5.88
Gunst GD et al. 2021 [34]	Camostat-mesilate	8	137	5.84
Gunst GD et al. 2021 [34]	Placebo	4	68	5.88
Gupta A et al. 2021 [35]	Sotrovimab	0	291	0
Gupta A et al. 2021 [35]	Placebo	1	292	0.34
Gutierrez-Castrellon P et al. 2021 [36]	NPF	0	150	0
Gutierrez-Castrellon P et al. 2021 [36]	Placebo	0	150	0
Jeronimo CMP et al. 2021 [39]	Methylprednisolone	72	194	37.11
Jeronimo CMP et al. 2021 [39]	Placebo	76	199	38.19
López-Medina E et al. 2021 [41]	Ivermectin	0	200	0
López-Medina E et al. 2021 [41]	Placebo	1	198	0.51
Marconi VC et al. 2021 [42]	Baricitinib	62	764	8.12
Marconi VC et al. 2021 [42]	Placebo	100	761	13.14
McCoy J et al. 2021 [43]	Proxalutamide	0	134	0
McCoy J et al. 2021 [43]	Placebo	2	128	1.56
Monk PD et al. 2020 [45]	IFN-β	0	50	0.00

Monk PD et al. 2020 [45]	Placebo	3	51	5.88
Patel O et al. 2021 [47]	HDIVZn	2	15	13.33
Patel O et al. 2021 [47]	Placebo	3	18	16.67
Puskarich MA et al. 2021 [48]	Losartan	0	58	0
Puskarich MA et al. 2021 [48]	Placebo	0	59	0
Ravikirti et al. 2021 [50]	Ivermectin	0	55	0
Ravikirti et al. 2021 [50]	Placebo	4	57	7.02
Salama C et al. 2021 [53]	Tocilizumab	26	249	10.44
Salama C et al. 2021 [53]	Placebo	11	128	8.59
Silva M et al. 2021 [54]	Nitazoxanide	1	27	3.70
Silva M et al. 2021 [54]	Placebo	1	13	7.69
Sivapalan P et al. 2021 [55]	HCQ/AZM	9	61	14.75
Sivapalan P et al. 2021 [55]	Placebo	6	56	10.71
Skipper CP et al. 2020 [56]	HCQ	1	212	0.47
Skipper CP et al. 2020 [56]	Placebo	1	211	0.47
Stone JH et al. 2020 [57]	Tocilizumab	9	161	5.59
Stone JH et al. 2020 [57]	Placebo	4	82	4.88
Tardif JC et al. 2021 [58]	Colchicine	5	2235	0.22
Tardif JC et al. 2021 [58]	Placebo	9	2253	0.40
Tornling G et al. 2021 [59]	C21	1	51	1.96
Tornling G et al. 2021 [59]	Placebo	3	55	5.45
Ulrich RJ et al. 2020 [60]	HCQ	7	67	10.45
Ulrich RJ et al. 2020 [60]	Placebo	6	61	9.84
Vallejos J et al. 2021 [61]	Ivermectin	4	250	1.60
Vallejos J et al. 2021 [61]	Placebo	3	251	1.20
Wang Y et al. 2020 [62]	Remdesivir	22	150	14.67
Wang Y et al. 2020 [62]	Placebo	10	77	12.99

Abbreviations: COVID-19: coronavirus disease 2019; LCP: low dosage CT-P59 (i.e., a monoclonal antibody with potent neutralizing activity); HCP: high dosage CT-P59; CPC: CT-P59 combined; HCQ: hydroxychloroquine; AZM: azithromycin; HDIVZn: high-dose intravenous zinc; IFN-β: interferon beta; NPF: novel probiotic formulation.

Supplementary Table 6. Included studies of network meta-analysis for treatment-emergent adverse events in non-severe patients with COVID-19.

Study (reference)	Intervention and control groups	Events	N	Ratio of treatment-emergent adverse events (%)
ACTIV-3/TICO LY-CoV555 Study Group 2021 [18]	LY-CoV555	38	163	23.31
ACTIV-3/TICO LY-CoV555 Study Group 2021 [18]	Placebo	30	151	19.87
Biber A et al. 2021 [21]	Ivermectin	2	47	4.26
Biber A et al. 2021 [21]	Placebo	3	42	7.14
Blum VF et al. 2021 [22]	Nitazoxanide	8	25	32.00
Blum VF et al. 2021 [22]	Placebo	13	25	52.00
Cadegiani FA et al. 2021 [24]	Proxalutamide	109	317	34.38
Cadegiani FA et al. 2021 [24]	Placebo	225	328	68.60
Chaccour C et al. 2021 [26]	Ivermectin	5	12	41.67
Chaccour C et al. 2021 [26]	Placebo	5	12	41.67
Chen J et al. 2020 [27]	LPV/r	9	52	17.31
Chen J et al. 2020 [27]	Arbidol	3	34	8.82
Chen J et al. 2020 [27]	Placebo	4	48	8.33
Dubee V et al. 2021 [29]	HCQ	2	124	1.61
Dubee V et al. 2021 [29]	Placebo	2	123	1.63
Eom JS et al. 2021 [30]	LCP	31	105	29.52
Eom JS et al. 2021 [30]	HCP	27	110	24.55
Eom JS et al. 2021 [30]	Placebo	34	110	30.91
Feld JJ et al. 2021 [31]	PL	2	30	6.67
Feld JJ et al. 2021 [31]	Placebo	1	30	3.33
Gonzalez-Ochoa AG et al. 2021 [32]	Sulodexide	96	124	77.42
Gonzalez-Ochoa AG et al. 2021 [32]	Placebo	85	119	71.43
Gottlieb RL et al. 2021 [33]	LDB	27	101	26.73
Gottlieb RL et al. 2021 [33]	MDB	26	107	24.30
Gottlieb RL et al. 2021 [33]	HDB	22	101	21.78
Gottlieb RL et al. 2021 [33]	MDB/etesevimab	19	112	16.96
Gottlieb RL et al. 2021 [33]	Placebo	42	156	26.92
Gunst GD et al. 2021 [34]	Camostat-mesilate	53	137	38.69
Gunst GD et al. 2021 [34]	Placebo	35	68	51.47
Gupta A et al. 2021 [35]	Sotrovimab	73	430	16.98
Gupta A et al. 2021 [35]	Placebo	85	438	19.41
Gutierrez-Castrellon P et al. 2021 [36]	NPF	41	150	27.33
Gutierrez-Castrellon P et al. 2021 [36]	Placebo	63	150	42.00
Humeniuk R et al. 2020 [37]	Remdesivir	17	78	21.79
Humeniuk R et al. 2020 [37]	Placebo	2	18	11.11
Jagannathan P et al. 2021 [38]	PL	36	60	60.00
Jagannathan P et al. 2021 [38]	Placebo	30	60	50.00
Lenze EJ et al. 2020 [40]	Fluvoxamine	12	80	15.00
Lenze EJ et al. 2020 [40]	Placebo	11	72	15.28
López-Medina E et al. 2021 [41]	Ivermectin	154	200	77.00
López-Medina E et al. 2021 [41]	Placebo	161	198	81.31
Marconi VC et al. 2021 [42]	Baricitinib	334	750	44.53
Marconi VC et al. 2021 [42]	Placebo	334	752	44.41

McCoy J et al. 2021 [43]	Proxalutamide	82	134	61.19
McCoy J et al. 2021 [43]	Placebo	116	128	90.63
Mohan A et al. 2020 [44]	Ivermectin	6	51	11.76
Mohan A et al. 2020 [44]	LDI	8	49	16.33
Mohan A et al. 2020 [45]	Placebo	6	52	11.54
Monk PD et al. 2020 [45]	IFN-β	26	48	54.17
Monk PD et al. 2020 [45]	Placebo	30	50	60.00
Reis G et al. 2021 [51]	HCQ	46	207	22.22
Reis G et al. 2021 [51]	LPV/r	92	232	39.66
Reis G et al. 2021 [51]	Placebo	46	220	20.91
Rocco PRM et al. 2020 [52]	Nitazoxanide	60	194	30.93
Rocco PRM et al. 2020 [52]	Placebo	60	198	30.30
Salama C et al. 2021 [53]	Tocilizumab	127	250	50.80
Salama C et al. 2021 [53]	Placebo	67	127	52.76
Silva M et al. 2021 [54]	Nitazoxanide	7	27	25.93
Silva M et al. 2021 [54]	Placebo	2	13	15.38
Skipper CP et al. 2020 [56]	HCQ	92	212	43.40
Skipper CP et al. 2020 [56]	Placebo	46	211	21.80
Stone JH et al. 2020 [57]	Tocilizumab	80	161	49.69
Stone JH et al. 2020 [57]	Placebo	46	82	56.10
Tardif JC et al. 2021 [58]	Colchicine	108	2195	4.92
Tardif JC et al. 2021 [58]	Placebo	139	2217	6.27
Ulrich RJ et al. 2020 [60]	HCQ	38	67	56.72
Ulrich RJ et al. 2020 [60]	Placebo	36	61	59.02
Vallejos J et al. 2021 [61]	Ivermectin	45	250	18.00
Vallejos J et al. 2021 [61]	Placebo	53	251	21.12
Wang Y et al. 2020 [62]	Remdesivir	102	155	65.81
Wang Y et al. 2020 [62]	Placebo	50	78	64.10
Weinreich DM et al. 2021 [63]	REGN-COV2	5	176	2.84
Weinreich DM et al. 2021 [63]	Placebo	7	93	7.53

Abbreviations: COVID-19: coronavirus disease 2019; LPV/r: lopinavir–ritonavir; PL: peginterferon lambda; LDI: low dosage ivermectin; LCP: low dosage CT-P59 (i.e., a monoclonal antibody with potent neutralizing activity); HCP: high dosage CT-P59; HCQ: hydroxychloroquine; LDB: low dosage bamlanivimab; MDB: moderate dosage bamlanivimab; HDB: high dosage bamlanivimab; IFN-β: interferon beta; NPF: novel probiotic formulation.

Supplementary Table 7. Included studies of network meta-analysis for all-cause mortality in severe patients with COVID-19.

Study (reference)	Intervention and control groups	Events	N	All-cause mortality (%)
CaoY et al. 2021 [64]	Ruxolitinib	0	20	0.00
CaoY et al. 2021 [64]	Placebo	3	21	14.29
Cremer PC et al. 2021 [65]	Mavrilimumab	1	21	4.76
Cremer PC et al. 2021 [65]	Placebo	3	19	15.79
de Alencar JCG et al. 2021 [66]	N-acetylcysteine	9	67	13.43
de Alencar JCG et al. 2021 [66]	Placebo	9	68	13.24
Dequin PF et al. 2020 [67]	Hydrocortisone	11	76	14.47
Dequin PF et al. 2020 [67]	Placebo	20	73	27.40
Gharebaghi N et al. 2020 [68]	IG	6	30	20.00
Gharebaghi N et al. 2020 [68]	Placebo	14	29	48.28
Lescure FX et al. 2021 [69]	Placebo	7	84	8.33
Lescure FX et al. 2021 [69]	LS	16	159	10.06
Lescure FX et al. 2021 [69]	HS	14	173	8.09
Libster R et al. 2021 [70]	CP	2	80	2.50
Libster R et al. 2021 [70]	Placebo	4	80	5.00
Munch MW et al. 2021 [71]	Hydrocortisone	6	16	37.50
Munch MW et al. 2021 [71]	Placebo	2	14	14.29
Rosas IO et al. 2021 [72]	Tocilizumab	58	294	19.73
Rosas IO et al. 2021 [72]	Placebo	28	144	19.44
Sehgal IS et al. 2021 [73]	Mycobacterium-w	4	20	20.00
Sehgal IS et al. 2021 [73]	Placebo	5	22	22.73
Simonovich VA et al. 2020 [75]	CP	25	228	10.96
Simonovich VA et al. 2020 [75]	Placebo	12	105	11.43
Sivapalasingam S et al. 2021 [76]	LS	60	242	24.79
Sivapalasingam S et al. 2021 [76]	HS	103	338	30.47
Sivapalasingam S et al. 2021 [76]	Placebo	43	170	25.29
Temesgen Z et al. 2021 [77]	Lenzilumab	23	236	9.75
Temesgen Z et al. 2021 [77]	Placebo	34	243	13.99
Zhong M et al. 2020 [78]	ALA	3	8	37.50
Zhong M et al. 2020 [78]	Placebo	7	9	77.78

Abbreviations: COVID-19: coronavirus disease 2019; CP: convalescent plasma; ALA: α-Lipoic acid; LS: low dosage sarilumab; HS: high dosage sarilumab; IG: immunoglobulin gamma.

Supplementary Table 8. Included studies of network meta-analysis for treatment-emergent adverse events in severe patients with COVID-19.

Study (reference)	Intervention and control groups	Events	N	Ratio of treatment-emergent adverse events (%)
CaoY et al. 2021 [64]	Ruxolitinib	7	20	35.00
CaoY et al. 2021 [64]	Placebo	6	21	28.57
Lescure FX et al. 2021 [69]	LS	103	159	64.78
Lescure FX et al. 2021 [69]	HS	121	173	69.94
Lescure FX et al. 2021 [69]	Placebo	55	84	65.48
Rosas IO et al. 2021 [72]	Tocilizumab	228	295	77.29
Rosas IO et al. 2021 [72]	Placebo	116	143	81.12
Sehgal IS et al. 2021 [73]	Mycobacterium-w	0	20	0.00
Sehgal IS et al. 2021 [73]	Placebo	0	22	0.00
Shi L et al. 2021 [74]	UC-MSCs	37	65	56.92
Shi L et al. 2021 [74]	Placebo	21	35	60.00
Simonovich VA et al. 2020 [75]	CP	153	228	67.11
Simonovich VA et al. 2020 [75]	Placebo	66	105	62.86
Sivapalasingam S et al. 2021 [76]	LS	19	50	38.00
Sivapalasingam S et al. 2021 [76]	HS	25	51	49.02
Sivapalasingam S et al. 2021 [76]	Placebo	7	25	28.00
Temesgen Z et al. 2021 [77]	Lenzilumab	68	255	26.67
Temesgen Z et al. 2021 [77]	Placebo	84	257	32.68

Abbreviations: COVID-19: coronavirus disease 2019; CP: convalescent plasma; LS: low dosage sarilumab; HS: high dosage sarilumab.

Supplementary Table 9. Assessment of incoherence for each outcome from the node-splitting model.

Evaluation of inconsistency using loop-specific heterogeneity estimates: A

Loop	IF	seIF	*z*_value	*p*_value	CI_95	Loop_Heterog_tau2
09-10-19	1.453	1.212	1.199	0.231	(0.00, 3.83)	0.277
09-13-19	0.132	1.356	0.098	0.922	(0.00, 2.79)	0.595
05-06-19	0.015	0.467	0.033	0.974	(0.00, 0.93)	0.000
03-04-11	–	–	–	–		0.000
03-04-19	–	–	–	–		0.000
07-12-19	–	–	–	–		0.000
07-15-19	–	–	–	–		0.000
07-14-15	–	–	–	–		0.000
07-14-19	–	–	–	–		0.000
07-12-15	–	–	–	–		0.000
12-14-15	–	–	–	–		0.000
12-15-19	–	–	–	–		0.000
12-14-19	–	–	–	–		0.000
03-11-19	–	–	–	–		0.000
04-11-19	–	–	–	–		0.000
07-12-14	–	–	–	–		0.000

A. The ratio of virological cure for non-severe COVID-19 patients (03: CT-P59 combined high dosage CT-P59; 05: hydroxychloroquine; 06: hydroxychloroquine/azithromycin; 07 high dosage bamlanivimab; 09: ivermectin; 10: ivermectin/ doxycycline; 11: low dosage CT-P59; 12: low dosage bamlanivimab; 13: low dosage ivermectin; 14: moderate dosage bamlanivimab; 15: moderate dosage bamlanivimab/etesevimab; 19: placebo).

Evaluation of inconsistency using loop-specific heterogeneity estimates: B

Loop	IF	seIF	*z*_value	*p*_value	CI_95	Loop_Heterog_tau2
03-07-14	–	–	–	–		0.000
03-14-20	–	–	–	–		0.000
03-07-20	–	–	–	–		0.000
07-14-20	–	–	–	–		0.000

B. All-cause mortality for non-severe COVID-19 patients (03: CT-P59 combined; 07: high dosage CT-P59 ; 14: low dosage CT-P59; 20: placebo).

Evaluation of inconsistency using loop-specific heterogeneity estimates: C

Loop	IF	seIF	*z*_value	*p*_value	CI_95	Loop_Heterog_tau2
07-14-21	0.407	0.814	0.500	0.617	(0.00, 2.00)	0.258
10-13-21	0.250	0.838	0.299	0.765	(0.00, 1.89)	0.000
01-14-21	0.077	1.088	0.070	0.944	(0.00, 2.21)	0.000
08-12-17	–	–	–	–		0.000
08-12-21	–	–	–	–		0.000
08-12-16	–	–	–	–		0.000
08-16-17	–	–	–	–		0.000
08-16-21	–	–	–	–		0.000
12-16-21	–	–	–	–		0.000
08-17-21	–	–	–	–		0.000
12-16-17	–	–	–	–		0.000

C. The ratio of treatment-emergent adverse events for non-severe COVID-19 patients (01: arbidol; 07: high dosage CT-P59; 08: hydroxychloroquine; 10: interferon beta; 12: low dosage CT-P59; 13: low dosage bamlanivimab; 14: low dosage ivermectin; 16: LY-CoV555; 17: moderate dosage bamlanivimab; 21: peginterferon lambda).

Evaluation of inconsistency using loop-specific heterogeneity estimates: D

Loop	IF	seIF	*z*_value	*p*_value	CI_95	Loop_Heterog_tau2
03-06-11	–	–	–	–		0.000

D. All-cause mortality of severe COVID-19 patients (03: high dosage sarilumab; 06: low dosage sarilumab; 11: placebo).

Evaluation of inconsistency using loop-specific heterogeneity estimates: E

Loop	IF	seIF	*z*_value	*p*_value	CI_95	Loop_Heterog_tau2
2-3-6	–	–	–	–		0.000

E. The ratio of treatment-emergent adverse events in severe COVID-19 patients (2: high dosage sarilumab; 3: low dosage sarilumab; 6: placebo).
