# Supplementary material for: Efficacy and safety of current medications for treating severe and non-severe COVID-19 patients: an updated network meta-analysis of randomized placebo-controlled trials
Source: Aging (Albany NY). 2021 Sep 16;13(18):21866–902. doi: 10.18632/aging.203522 (PMC8507270; doi:10.18632/aging.203522)
Supplement: Appendix 1 [file aging-13-203522-s005.docx]

**Appendix 1**

**Full search strategies:**

Through all fields of advanced, we used the following search terms or keywords alone or in combination: “coronavirus disease*” OR “COVID*” OR “coronavirus disease 19*” OR “COVID-19*” OR “SARS-CoV-2” OR “treatment*” OR “therapy*” OR “hydroxychloroquine*” OR “chloroquine*” OR “ivermectin*” OR “avifavir*” OR “doxycycline*” OR “sarilumab*” OR “colchicine*” OR “interferon*” OR “lopinavir/ritonavir*” OR “convalescent plasma*” OR “arbidol*” OR “remdesivir*” OR “standard of care*” OR “α-Lipoic acid*” OR “monoclonal antibody*” OR “auxora*” OR “tocilizumab*” OR “hormone*” OR “ayurvedic*” OR “nitazoxanide*” OR “lenzilumab*”OR “hydrocortisone*” OR “imatinib*” OR “ruxolitinib*” OR “baricitinib*” OR “proxalutamide*”, of which strategies were mainly divided into two parts (severe and non-severe COVID-19 infections).
