# Supplementary material for: Efficacy and safety of current medications for treating severe and non-severe COVID-19 patients: an updated network meta-analysis of randomized placebo-controlled trials
Source: Aging (Albany NY). 2021 Sep 16;13(18):21866–902. doi: 10.18632/aging.203522 (PMC8507270; doi:10.18632/aging.203522)
Supplement: Appendix 2 [file aging-13-203522-s006.docx]

**Appendix 2**

**The network meta-analysis program of STATA software for the ratio of virological cure in non-severe patients with COVID-19**

*network setup r n, stud(study) trt(t) num ref(Placebo)*

*network map, improve*

*network meta i*

*network meta consistency*

*network sidesplit all, tau*

*network rank min, all zero reps(5000) gen(prob)*

*sucra prob*, lab(Placebo Arbidol Ayurvedic CPC HCP HCQ HCQ/AZM HDB HDVD Ivermectin Ivermectin/Doxycycline LCP LDB LDI MDB MDB/Etesevimab Methylprednisolone Nitazoxanide PL Proxalutamide REGN-COV2 Remdesivir)*

*netleague, lab(Placebo Arbidol Ayurvedic CPC HCP HCQ HCA HDB HDVD Ivermectin ID LCP LDB LDI MDB MDE Methylprednisolone Nitazoxanide PL Proxalutamide RC Remdesivir) sort(Ayurvedic Proxalutamide HDVD Ivermectin LCP ID PL Nitazoxanide CPC Arbidol RC HCP LDI HCA Remdesivir MDE LDB Methylprednisolone MDB Placebo HDB HCQ) eform*

*network convert pairs*

*netfunnel _y _stderr _t1 _t2 , random bycomp add(lfit _stderr _ES_CEN) noalpha*

*ifplot _y _stderr _t1 _t2 study, tau2(loop)*

*midas tp fp fn tn,reg(ms cd ss ds is id db rrb lci)*

**The network meta-analysis program of STATA software for all-cause mortality in non-severe patients with COVID-19**

*network setup r n, stud(study) trt(t) num ref(Placebo)*

*network map, improve*

*network meta i*

*network meta consistency*

*network sidesplit all, tau*

*network rank min, all zero reps(5000) gen(prob)*

*sucra prob*, lab(Placebo Baricitinib C21 CPC Camostat-mesilate Canakinumab Colchicine HCP HCQ HCQ/AZM HDIVZn IFN-β Imatinib Ivermectin LCP LY-CoV555 Losartan Methylprednisolone NPF Nitazoxanide Proxalutamide Remdesivir Sotrovimab Sulodexide Tocilizumab)*

*netleague, lab(Placebo Baricitinib C21 CPC CM Canakinumab Colchicine HCP HCQ HCA HDIVZn IFNβ Imatinib Ivermectin LCP LC Losartan Methylprednisolone NPF Nitazoxanide Proxalutamide Remdesivir Sotrovimab Sulodexide Tocilizumab) sort(Proxalutamide IFNβ Nitazoxanide Sulodexide C21 Imatinib Sotrovimab Baricitinib Colchicine Canakinumab HCQ HDIVZn Ivermectin NPF Losartan Methylprednisolone CM Placebo Remdesivir Tocilizumab HCA LC HCP CPC LCP) eform*

*network convert pairs*

*netfunnel _y _stderr _t1 _t2 , random bycomp add(lfit _stderr _ES_CEN) noalpha*

*ifplot _y _stderr _t1 _t2 study, tau2(loop)*

*midas tp fp fn tn,reg(ms cd ss ds is id db rrb lci)*

**The network meta-analysis program of STATA software for the rate of treatment-emergent adverse events in non-severe patients with COVID-19**

*network setup r n, stud(study) trt(t) num ref(Placebo)*

*network map, improve*

*network meta i*

*network meta consistency*

*network sidesplit all, tau*

*network rank min, all zero reps(5000) gen(prob)*

*ssucra prob*, lab(Placebo Arbidol Baricitinib CPC Camostat-mesilate Colchicine Fluvoxamine HCP HCQ HDB IFN-β Ivermectin LCP LDB LDI LPV/r LY-CoV555 MDB MDB/Etesevimab NPF Nitazoxanide PL Proxalutamide REGN-COV2 Remdesivir Sotrovimab Sulodexide Tocilizumab)*

*netleague, lab(Placebo Arbidol Baricitinib CPC CM Colchicine Fluvoxamine HCP HCQ HDB IFNβ Ivermectin LCP LDB LDI LPVr LC MDB MDE NPF Nitazoxanide PL Proxalutamide RC Remdesivir Sotrovimab Sulodexide Tocilizumab) sort(Proxalutamide RC NPF MDE CM HCP HDB Colchicine Ivermectin IFNβ CPC Tocilizumab Sotrovimab Nitazoxanide MDB LCP Fluvoxamine Arbidol LDB Baricitinib Placebo HCQ LC Remdesivir LDI Sulodexide PL LPVr) eform*

*network convert pairs*

*netfunnel _y _stderr _t1 _t2 , random bycomp add(lfit _stderr _ES_CEN) noalpha*

*ifplot _y _stderr _t1 _t2 study, tau2(loop)*

*midas tp fp fn tn,reg(ms cd ss ds is id db rrb lci)*

**The network meta-analysis program of STATA software for all-cause mortality in severe patients with COVID-19**

*network setup r n, stud(study) trt(t) num ref(Placebo)*

*network map, improve*

*network meta i*

*network meta consistency*

*network sidesplit all, tau*

*network rank min, all zero reps(5000) gen(prob)*

*sucra prob*, lab(Placebo ALA CP HS Hydrocortisone IG LS Lenzilumab Mavrilimumab Mycobacterium-w N-acetylcysteine Ruxolitinib Tocilizumab)*

*netleague, lab(Placebo ALA CP HS Hydrocortisone IG LS Lenzilumab Mavrilimumab Mycobacteriumw Nacetylcysteine Ruxolitinib Tocilizumab) sort(ALA IG Ruxolitinib Mavrilimumab Lenzilumab Hydrocortisone CP Mycobacteriumw Nacetylcysteine Placebo Tocilizumab LS HS) eform*

*network convert pairs*

*netfunnel _y _stderr _t1 _t2 , random bycomp add(lfit _stderr _ES_CEN) noalpha*

*ifplot _y _stderr _t1 _t2 study, tau2(loop)*

*midas tp fp fn tn,reg(ms cd ss ds is id db rrb lci)*

**The network meta-analysis program of STATA software for the rate of treatment-emergent adverse events in severe patients with COVID-19**

*network setup r n, stud(study) trt(t) num ref(Placebo)*

*network map, improve*

*network meta i*

*network meta consistency*

*network sidesplit all, tau*

*network rank min, all zero reps(5000) gen(prob)*

*sucra prob*, lab(Placebo CP HS LS Lenzilumab Mycobacterium-w Ruxolitinib Tocilizumab UC-MSCs)*

*netleague, lab(Placebo CP HS LS Lenzilumab Mycobacteriumw Ruxolitinib Tocilizumab UCMSCs) sort(Mycobacteriumw HS Ruxolitinib CP Placebo UCMSCs LS Tocilizumab Lenzilumab) eform*

*network convert pairs*

*netfunnel _y _stderr _t1 _t2 , random bycomp add(lfit _stderr _ES_CEN) noalpha*

*ifplot _y _stderr _t1 _t2 study, tau2(loop)*

*midas tp fp fn tn,reg(ms cd ss ds is id db rrb lci)*
